# Fungal extracellular vesicles reshape *Lactobacillus rhamnosus* vesicles to enhance lysosomal homeostasis and resolve intestinal inflammation

**DOI:** 10.1080/19490976.2026.2736915

**Published:** 2026-09-19

**Authors:** Zhengmei Ji, Guoliang Chen, Xiaolong He, Xiangqian Zhang, Bin Chen, Hang Xiao, Yurong Guo, Yanhui Han

**Affiliations:** a Engineering Research Center for High-Valued Utilization of Fruit Resources in Western China, Ministry of Education, Shaanxi Normal University, Changan, Xi'an, PR China; b National Research & Development Center of Apple Processing Technology, Shaanxi Normal University, Changan Xi'an, PR China; c College of Food Engineering and Nutritional Science, Shaanxi Normal University, Changan Xi'an, PR China; d Research and Development Centre of Ecological and Sustainable application of Microbial Industry of the Loess Plateau in Shaanxi Province, College of Life Science, Yan'an University, Yan'an Shaanxi, PR China; e Department of Food Science, University of Massachusetts, Amherst, Massachusetts, United States

**Keywords:** Extracellular vesicles, gut microbiota, *Lactobacillus*, hydroxylated ceramide, lysosome-autophagy, colitis

## Abstract

Extracellular vesicles (EVs) are emerging mediators of intercellular and interkingdom communication. While bacterial EVs directly modulate host health, whether exogenous vesicles can reprogram gut microbial vesiculation and bioactivity remains unclear. Using a defined *in vitro* conditioning system, ScEV exposure altered EV production by *Lactobacillus rhamnosus*, generating ScEV-conditioned *L. rhamnosus* vesicles (ScEV-LrEVs). Comparative proteomics and lipidomics revealed extensive remodeling of vesicle cargo, including ~60-fold enrichment of hydroxylated ceramide Cer(t18:0/24:0(2OH)) in ScEV-LrEVs. Functionally, ScEV-LrEVs showed enhanced anti-inflammatory activity in LPS-stimulated macrophages, accompanied by improved lysosomal function and coordinated changes in mTOR-TFEB-related lysosome-autophagy markers. In a DSS-induced colitis model, ScEV-LrEVs alleviated mucosal injury, reduced immune-cell infiltration, and reinforced epithelial barrier integrity, accompanied by restoration of lysosomal function and coordinated changes in mTOR-TFEB-related markers. Ceramide-enriched EVs partially recapitulated the anti-inflammatory and lysosome-restorative activity of ScEV-LrEVs. Together, these findings uncover a cross-kingdom vesicle communication axis whereby fungal EV exposure remodels the molecular and functional properties of gut microbial vesicles, accompanied by enhanced lysosomal homeostasis and protection against intestinal inflammation.

## Graphical abstract

**Figure:**
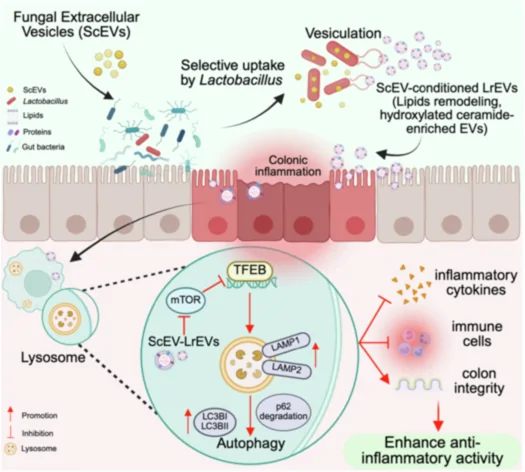

## 1. Introduction

Extracellular vesicles (EVs) have emerged as universal mediators of communication across biological kingdoms. These nanoscale lipid bilayer structures carry diverse bioactive cargos and regulate immunity, metabolism, and microbial ecology.^1^ Once considered cellular debris, EVs are now recognized as organized signaling entities that regulate immunity, metabolism, and microbial ecology.^2^ ^,^ ^3^ While mammalian EVs have been widely studied, recent findings reveal that plants and fungi also release EVs enriched in enzymes, lipids, and small RNAs capable of modulating microbial or host physiology. For instance, ginger-derived exosome-like nanoparticles can reprogram gut microbial metabolism and alleviate inflammation, highlighting the nutritional and therapeutic potential of food-derived vesicles.^4^ Edible and filamentous fungi secrete abundant EVs, which carry diverse signaling molecules such as polysaccharides, lipids, and immunomodulatory enzymes.^5^ In addition, fungal EVs contain vesicle-bound bioactive factors capable of influencing microbial activity and metabolite exchange,^6^ suggesting that fungi contribute actively to microbial network regulation through vesicular signaling. Although fungal EVs have been identified across diverse species, their molecular composition and biological functions, particularly in shaping gut microbial ecology and influencing host responses, remain poorly understood.

Within the gut, microbial EVs serve as a crucial communication interface between the bacteria and the host by regulating immune responses, maintaining epithelial homeostasis, and controlling inflammation.^7^ ^,^ ^8^ For instance, bacteria-derived EVs can traverse the mucus barrier and interact with epithelial and immune cells, thereby modulating cytokine networks, strengthening tight junctions, and alleviating barrier dysfunction.^9^ ^,^ ^10^ Moreover, probiotic-derived EVs can recapitulate the beneficial effects of their parent bacteria by attenuating colitis, balancing macrophage polarization, and restoring gut integrity.^11^ ^,^ ^12^ Accumulating evidence shows that plant- and fungus-derived EVs can enter the gastrointestinal tract and interact with gut microbiota, thereby altering bacterial metabolism, community structure, and the bioactivity of bacterial products. These findings raise the intriguing possibility that fungal EVs may modulate gut bacterial vesiculation and, in turn, influence host physiological responses. However, the mechanisms by which plant- or fungal-derived EVs govern gut microbial vesiculation and their biological activity remain largely undefined.

Intestinal inflammatory homeostasis is closely linked to lysosome-autophagy regulation, which supports cellular clearance and limits persistent inflammatory signaling.^13^ Dysregulation of lysosomal function contributes to defective cellular clearance and persistent inflammation in the gut.^14^ Recent studies suggest that microbial metabolites and vesicular lipids can influence these lysosome-autophagy processes, linking microbial signaling to host cell clearance mechanisms.^15^ ^,^ ^16^ However, direct evidence connecting microbial EVs to the regulation of lysosomal function remains limited, raising the question of whether vesicle-mediated communication can restore lysosomal homeostasis and promote inflammation resolution.

In this study, we explored how fungal EVs influence bacterial vesiculation and host inflammatory responses. We isolated extracellular vesicles from the edible filamentous fungus *Schizophyllum commune* (ScEVs) and investigated their interactions with gut microbiota. Using *in vivo* tracing and *in vitro* co-culture systems, we found that ScEVs were internalized by defined gut microbes and selectively interacted with specific gut bacteria. Short- and long-term exposure assays identified *Lactobacillus rhamnosus* as a primary gut microbial responder to ScEVs. Multi-omic analyzes revealed that ScEV conditioning was associated with extensive alterations in the proteomic and lipidomic profiles of *L. rhamnosus* EVs (ScEV-LrEVs), including marked enrichment of hydroxylated sphingolipids, particularly Cer(t18:0/24:0(2OH)). Functionally, ScEV-LrEVs alleviated colitis via restoring lysosomal function in cellular and murine models. Together, these findings establish an experimentally defined ScEV-conditioning model in which fungal EV exposure alters the molecular and functional properties of *L. rhamnosus* EVs, while the extent to which analogous conditioning occurs *in vivo* remains to be determined. This work expands the conceptual boundaries of vesicle-mediated communication in the gut, revealing how fungal-bacterial interactions can be harnessed to modulate gut health.

## 2. Results

### 2.1. Characterization and biodistribution of *Schizophyllum commune*-derived extracellular vesicles (ScEVs)

**Figure 1.**
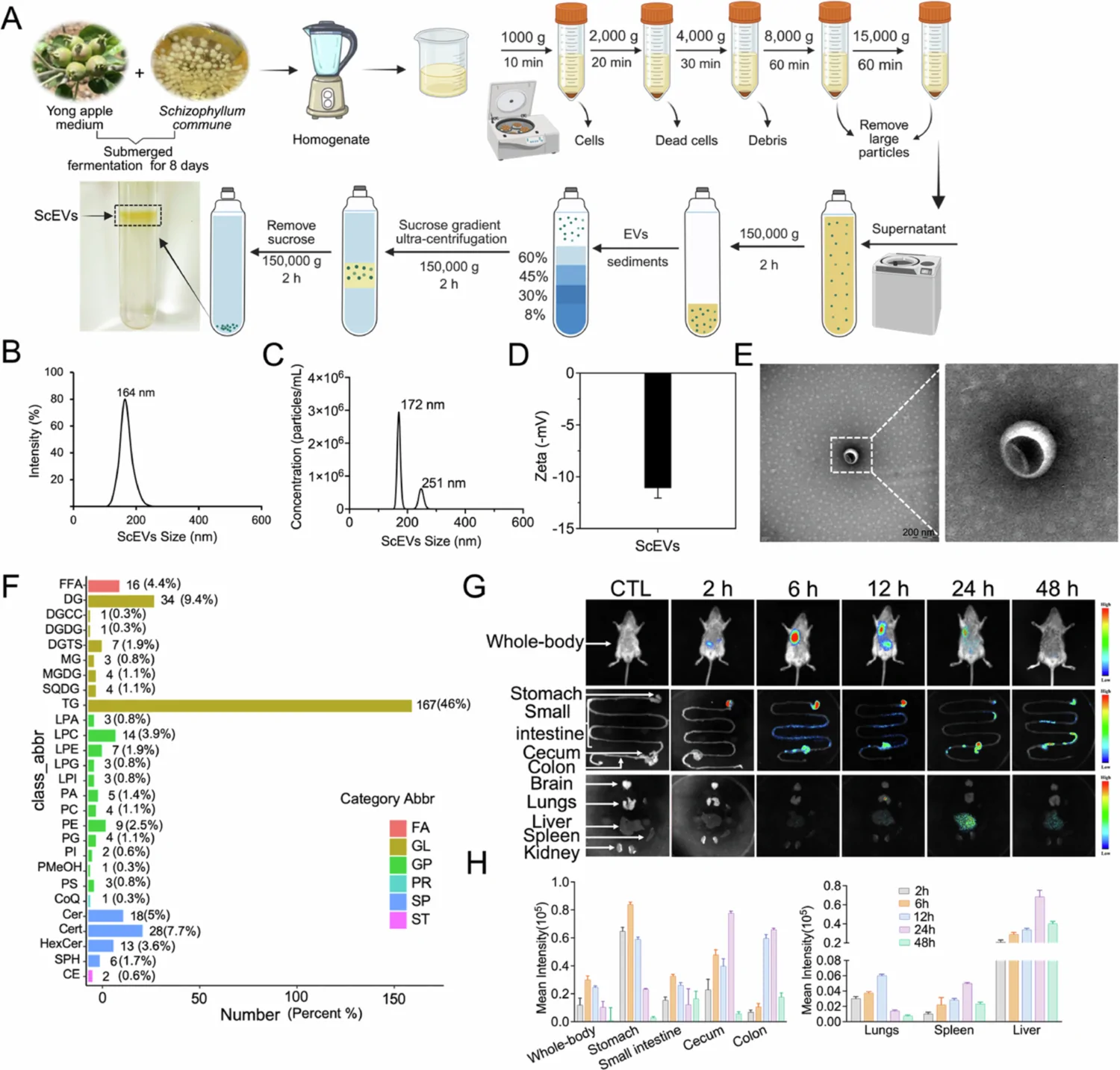
Isolation, characterization, and biodistribution of EVs from Schizophyllum commune (ScEVs). (A) Schematic of the isolation and purification process of ScEVs. (B–C) Particle size distribution of ScEVs measured by dynamic light scattering (DLS) and nanoparticle tracking analysis (NTA). Representative profiles are shown, and quantitative NTA values are presented as mean ± SD from three independent ScEV preparations, each measured in three technical replicates. Raw NTA replicate curves are provided in Fig. S1(A). (D) Zeta potential of ScEVs (*n* = 3 independent ScEV preparations). (E) Transmission electron microscopy image showing the typical cup-shaped morphology of ScEVs (scale bar, 200 nm). (F) Lipidomic signature of ScEVs (*n* = 3 independent ScEV preparations). (G) Representative *in vivo* and ex vivo fluorescence images showing the biodistribution of DiR-labeled ScEV-associated signals following oral administration in mice. A separate cohort of mice was used at each terminal time point (*n* = 6 mice per time point). (H) Quantification of fluorescence intensity in major organs and gastrointestinal regions (*n* = 6 mice per terminal time point). Quantitative data are presented as mean ± SD, and statistical analyzes were performed as described in the main methods.

To obtain extracellular vesicles secreted by *Schizophyllum commune* (ScEVs), the fungus was cultured under liquid fermentation using immature apple biomass as a sustainable substrate, as our previous work showed that *S. commune* efficiently valorizes fruit residues for secondary metabolite production.^17^ After eight days, ScEVs were isolated from the culture supernatant by sequential differential centrifugation and sucrose density-gradient ultracentrifugation (Figure 1A). Representative DLS and NTA profiles are shown in Figure 1B and Figure 1C, respectively, while the corresponding data from three independent biological preparations are provided in Fig. S1A and Fig. S1B. DLS analysis indicated a nanoscale size distribution with a mean diameter of 192 ± 49 nm, whereas NTA showed a mean particle diameter of 190 ± 84 nm and confirmed the presence of abundant nanosized vesicles. Zeta potential analysis demonstrated that ScEVs carried a negative surface charge (Figure 1D). Transmission electron microscopy (TEM) revealed the characteristic cup-shaped morphology of ScEVs (Figure 1E).

Lipidomic profiling revealed that ScEVs comprised multiple lipid classes, including glycerolipids (GL, 51.5%), sphingolipids (SP, 18%), and phospholipids (GP, 15.3%), with a notable fraction of ceramides and hexosylceramides (Figure 1F). Proteomic analysis identified 223 proteins and 857 peptides (Fig. S1C), predominantly annotated to metabolic and cellular processes (Fig. S1D). Clusters of Orthologous Groups (COG) classification revealed enrichment in metabolism-related functions, including carbohydrate, amino acid, and lipid metabolism, as well as cellular information processing and transport (Fig. S1E). Gene Ontology (GO) analysis indicated strong associations with catalytic activity, membrane transport, and oxidoreductase functions (Fig. S1D), while KEGG pathways highlighted lysosomal activity and glycosphingolipid biosynthesis (Fig. S1F). Together, these results indicate that ScEVs carry metabolically active biomolecules with potential signaling functions.

For biodistribution, DiR-labeled ScEVs were administered orally to mice and tracked by *in vivo* fluorescence imaging (Figure 1G). Representative fluorescence images showed that DiR-associated signals were predominantly distributed in the stomach and small intestine at 6 h, whereas more prominent signals were observed in the cecum and colon at 24 h (Figure 1H). At 24 h, a hepatic signal was also observed, suggesting partial translocation into systemic circulation. Overall, DiR-associated fluorescence signals displayed a time-dependent distribution along the gastrointestinal tract and were also detected in selected host tissues, supporting the intestinal accessibility of orally administered ScEVs under healthy conditions and their potential interactions with gut microbiota.

### 2.2. ScEVs associate with defined gut bacteria and increase net EV output from *Lactobacillus* cultures

**Figure 2.**
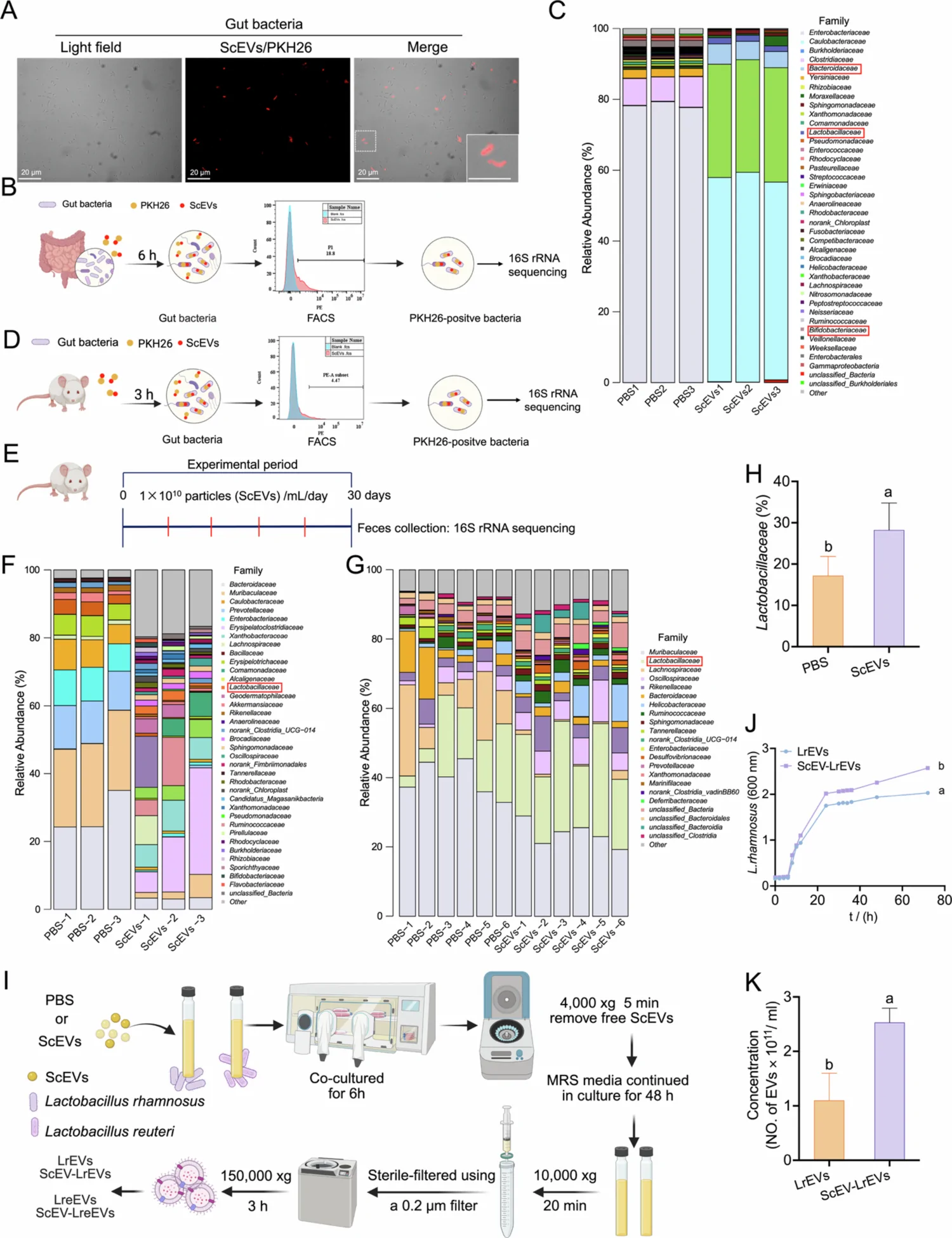
ScEVs associate with gut microbiota and increase net EV output from *Lactobacillus* cultures. (A) Confocal microscopy images showing ScEV-associated fluorescence in fecal bacteria (scale bar, 20 μm, representative images from *n* = 3 independent experiments). (B) Workflow for isolating ScEV-positive bacteria using fluorescence-activated cell sorting (FACS) followed by 16S rRNA gene sequencing. (C) Family-level taxonomic composition of ScEV-associated bacterial populations. Fecal samples from nine donors were pooled to generate three composite samples per group, with each composite sample comprising material from three donors (*n* = 3 composite samples per group). (D) Schematic of the short-term uptake experiment. Mice were orally gavaged with PKH26-labeled ScEVs (1 × 10^10^ particles/mL, 2 × 10^9^ particles per mouse) for 3 h, after which fecal bacteria were collected for FACS and sequencing. Each group contained nine mice, which generated three composite sequencing samples by pooling fecal samples from three mice (*n* = 9 mice and *n* = 3 composite sequencing samples per group). (E) Experimental design for the 30-day administration study. Mice received 200 μL of ScEV suspension (1 × 10^10^ particles/mL, corresponding to 2 × 10^9^ particles per mouse) by daily oral gavage for 30 consecutive days (*n* = 6 mice per group). (F) Family-level bacterial profiles of ScEV-positive and total bacterial communities after short-term ScEV exposure (*n* = 3 composite sequencing samples per group, each generated by pooling fecal samples from three mice). (G) Family-level bacterial profiles of ScEV-positive and total communities after long-term exposure to ScEVs (*n* = 6 per group). (H) Relative abundance of Lactobacillaceae in fecal samples from PBS- and ScEV-treated mice after long-term administration (*n* = 6 mice per group). (I) Workflow illustrating the coculture strategy used to generate ScEV-conditioned bacterial EVs. *Lactobacillus* cells were conditioned with ScEVs for 6 h, washed to remove free ScEVs, re-cultured in fresh MRS medium for 48 h, and then subjected to EV isolation. (J) Growth curves of *L. rhamnosus* with or without ScEV exposure (*n* = 3 independent experiments). (K) NTA quantification of EV yield from L. rhamnosus cultures. Bacterial cells were conditioned with ScEVs for 6 h, washed, transferred to fresh MRS medium, and cultured for an additional 48 h before EV isolation and quantification (*n* = 3 independent EV preparations). Quantitative data are presented as mean ± SD, and statistical analyzes were performed as described in the main methods.

Given their intestinal localization, we next investigated whether ScEVs directly interact with gut microbiota. PKH26-labeled ScEVs were exposed to human fecal samples for 6 h, followed by fluorescence imaging, which showed ScEV-associated fluorescent signals in association with bacterial cells (Figure 2A). ScEV-positive bacteria were isolated by fluorescence-activated cell sorting (FACS) and analyzed by 16S rRNA gene sequencing (Figure 2B). ScEV-positive fractions contained multiple commensal taxa, including *Bacteroidaceae*, *Bifidobacteriaceae*, and *Lactobacillaceae* (Figure 2C).

To validate these interactions *in vivo*, mice were orally administered PKH26-labeled ScEVs, and fecal samples were collected 3 h post-gavage for bacterial sorting and sequencing (Figure 2D). ScEV-associated bacterial populations mirrored the *in vitro* results, with enrichment in *Lactobacillaceae*, *Bacteroidaceae*, and *Bifidobacteriaceae* (Figure 2F). These data indicate that ScEV-associated fluorescent signals can be detected in multiple gut bacterial populations at an early time point following oral administration, supporting an early association between ScEVs and gut bacteria.

The effect of long-term exposure of ScEVs on gut microbiota was further investigated (Figure 2E). 16S rRNA sequencing revealed clear compositional shifts relative to controls, with *Lactobacillaceae* emerging as one of the most enriched families (Figure 2G and H). These results indicate that ScEV exposure is associated with multiple gut bacterial populations at an early time point and, following long-term administration, is accompanied by changes in intestinal microbial composition, including an increased relative abundance of *Lactobacillaceae*. To provide a broader taxonomic perspective, corresponding phylum- and genus-level profiles were also analyzed for the 6 h *in vitro* incubation, 3 h *in vivo* gavage, and 30-day administration experiments (Fig. S2A–F). These additional profiles further supported the consistent association of *Lactobacillus*-related taxa with ScEV exposure .

Although ScEVs were associated with multiple gut bacterial taxa, *Lactobacillus* was consistently detected among ScEV-positive bacterial populations in both *in vitro* fecal microbiota incubation and short-term *in vivo* gavage experiments, and its relative abundance was further increased after long-term ScEV administration. Therefore, *Lactobacillus* was selected as a representative responsive genus for strain-level validation. Both *L. rhamnosus* and *L. reuteri* showed ScEV-associated fluorescent signals following 6 h of incubation with PKH26-labeled ScEVs (Fig. S3A). ScEV exposure noticeably accelerated growth of *L. rhamnosus* and *L. reuteri* (Figures 2J and S3F). To generate ScEV-conditioned bacterial EVs, *L. rhamnosus* and *L. reuteri* were first co-incubated with ScEVs for 6 h as a conditioning step. Bacterial cells were then collected, washed to remove free ScEVs, transferred to fresh MRS medium, and cultured for an additional 48 h before EV isolation from the culture supernatant (Figure 2I). This workflow was used to minimize ScEV carryover and to assess EV production from ScEV-conditioned bacteria. ScEV conditioning was accompanied by a significant increase in the concentration of EV particles recovered from *L. rhamnosus* and *L. reuteri* cultures (Figs. 2 K and S3G), without altering their size distribution (Figs. S3B–E). Taken together, these results indicate that ScEV conditioning promotes *Lactobacillus* growth and is associated with increased net EV output from the resulting bacterial cultures.

### 2.3. ScEV-conditioned bacterial vesicles enhance anti-inflammatory responses associated with restoration of lysosomal function

We next investigated whether ScEV conditioning alters the inflammatory response of *Lactobacillus*-derived EVs. Confocal microscopy confirmed efficient uptake of both native and ScEV-conditioned EVs by RAW264.7 macrophages within 6 h (Fig. S4A). Cytotoxicity assays showed no adverse effects across a wide concentration range (10^6^−10^11^ particles/mL) (Figs. S4B and S4C).

In LPS-stimulated macrophages, both LrEVs and LreEVs significantly reduced nitric oxide, IL-6, TNF-*α*, IL-1β, and IL-4 production, while increasing the anti-inflammatory cytokine IL-10 (Fig. S4D). Importantly, ScEV-conditioned vesicles (ScEV-LrEVs and ScEV-LreEVs) exhibited a stronger inhibitory effect on pro-inflammatory mediators, particularly NO, IL-6, and TNF-*α*, suggesting that fungal vesicle conditioning enhances the anti-inflammatory potential of *Lactobacillus* EVs. Furthermore, ROS measurements showed that EV treatment, particularly with ScEV-LrEVs, significantly suppressed LPS-induced oxidative stress (Figs. S5A and S5B). Collectively, these results demonstrate that ScEV conditioning augments the anti-inflammatory activity of *Lactobacillus*-derived EVs.

**Figure 3.**
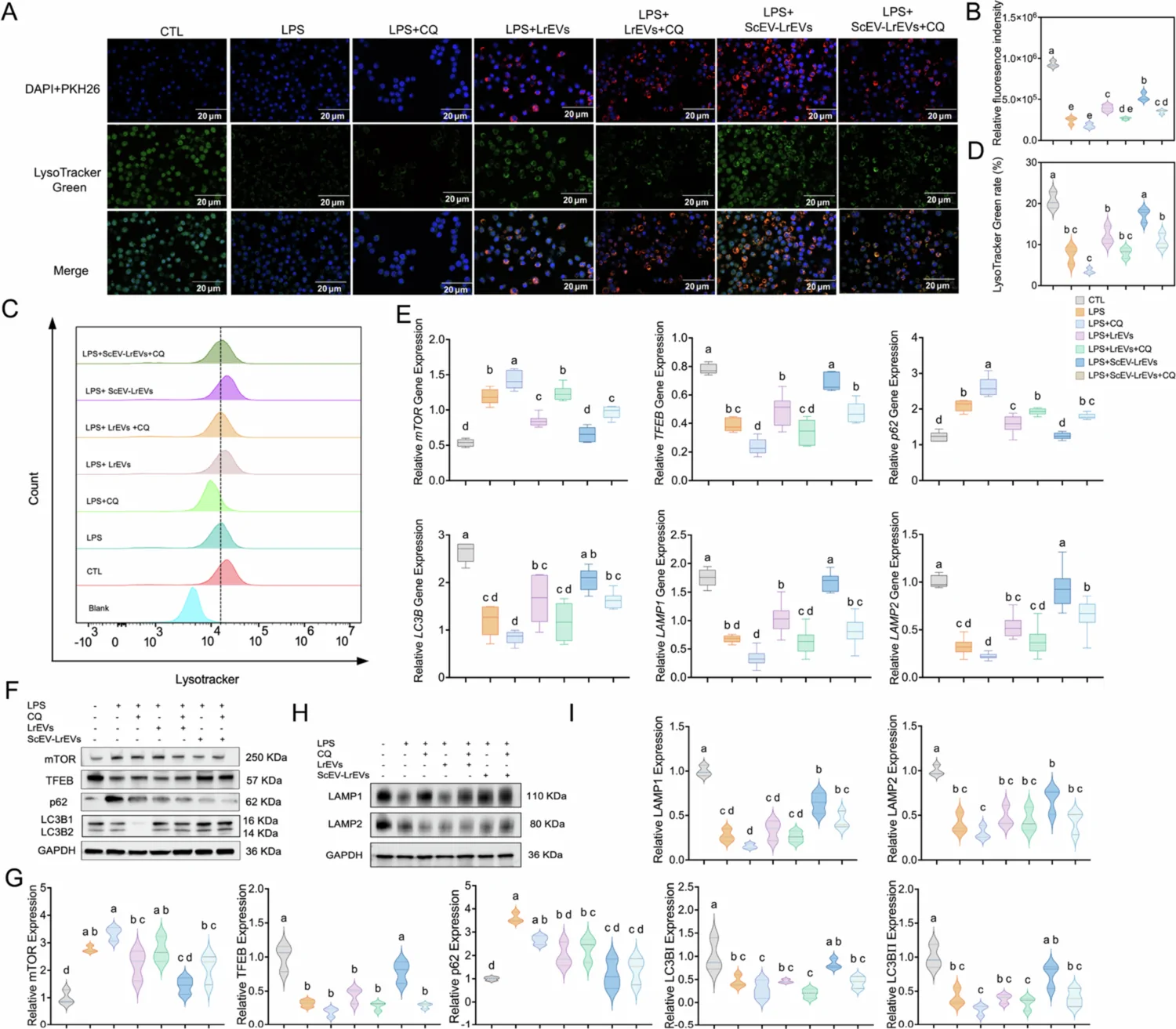
ScEV-conditioning enhances the anti-inflammatory activity of *L. rhamnosus* EVs in association with the mTOR-TFEB-lysosome axis. (A) Representative confocal images of RAW264.7 macrophages stained with LysoTracker Green after treatment with LrEVs or ScEV-LrEVs, with or without chloroquine (CQ, 20 μM). Lysosomal activity is visualized by LysoTracker fluorescence (scale bar, 20 μm, representative images from *n* = 3 independent experiments). (B) Quantification of lysosomal fluorescence intensity from confocal microscopy images (*n* = 3 independent experiments). (C) Flow cytometry analysis of LysoTracker fluorescence showing lysosomal activity under different EV treatments (*n* = 3 independent experiments). (D) Quantification of LysoTracker mean fluorescence intensity (MFI) from flow cytometry (*n* = 3 independent experiments). (E) Relative mRNA expression levels of *mTOR*, *TFEB*, *p62*, *LC3B*, *LAMP1*, and *LAMP2* in RAW264.7 cells after EV treatment, assessed by qRT-PCR (*n* = 3 independent experiments). (F−G) Western blot analysis of mTOR, TFEB, p62, LC3B-I/II, and GAPDH (*n* = 3 independent experiments). (H−I) Western blot analysis and quantification of lysosomal membrane proteins LAMP1 and LAMP2 (*n* = 3 independent experiments). Quantitative data are presented as mean ± SD, and statistical analyzes were performed as described in the main methods.

Because *L. rhamnosus* displayed stronger anti-inflammatory potency, it was selected for subsequent mechanistic investigation. LysoTracker staining revealed that LPS impaired lysosomal function, which was restored by both LrEVs and ScEV-LrEVs, with the latter showing the strongest effect (Figure 3A−D). The inclusion of the lysosomal inhibitor chloroquine (CQ) largely abolished this recovery, suggesting that lysosomal function contributes to the anti-inflammatory activity of ScEV-LrEVs.

At the molecular level, both LrEVs and ScEV-LrEVs significantly downregulated the transcription of *mTOR* while upregulating *TFEB* expression compared with the LPS group, with the most pronounced transcriptional changes observed in the ScEV-LrEVs-treated cells. In parallel, *p62* mRNA levels were markedly decreased, whereas *LC3B*, *LAMP1*, and *LAMP2* transcripts were substantially increased, indicating coordinated upregulation of lysosome- and autophagy-related transcripts (Figure 3E). Consistent with these transcriptional alterations, Western blot analysis further confirmed the corresponding protein-level changes. ScEV-LrEVs treatment resulted in a pronounced reduction of phosphorylated mTOR and p62 accumulation, together with elevated levels of TFEB, LC3B-I, LC3B-II, LAMP1, and LAMP2 proteins (Figures 3F−I). Collectively, these results indicate that ScEV conditioning enhances the ability of *Lactobacillus*-derived EVs to restore lysosomal function and coordinate lysosome- and autophagy-related molecular responses, which is accompanied by improved anti-inflammatory activity.

### 2.4. ScEV-conditioned *Lactobacillus* vesicles alleviate colitis and restore epithelial barrier integrity

**Figure 4.**
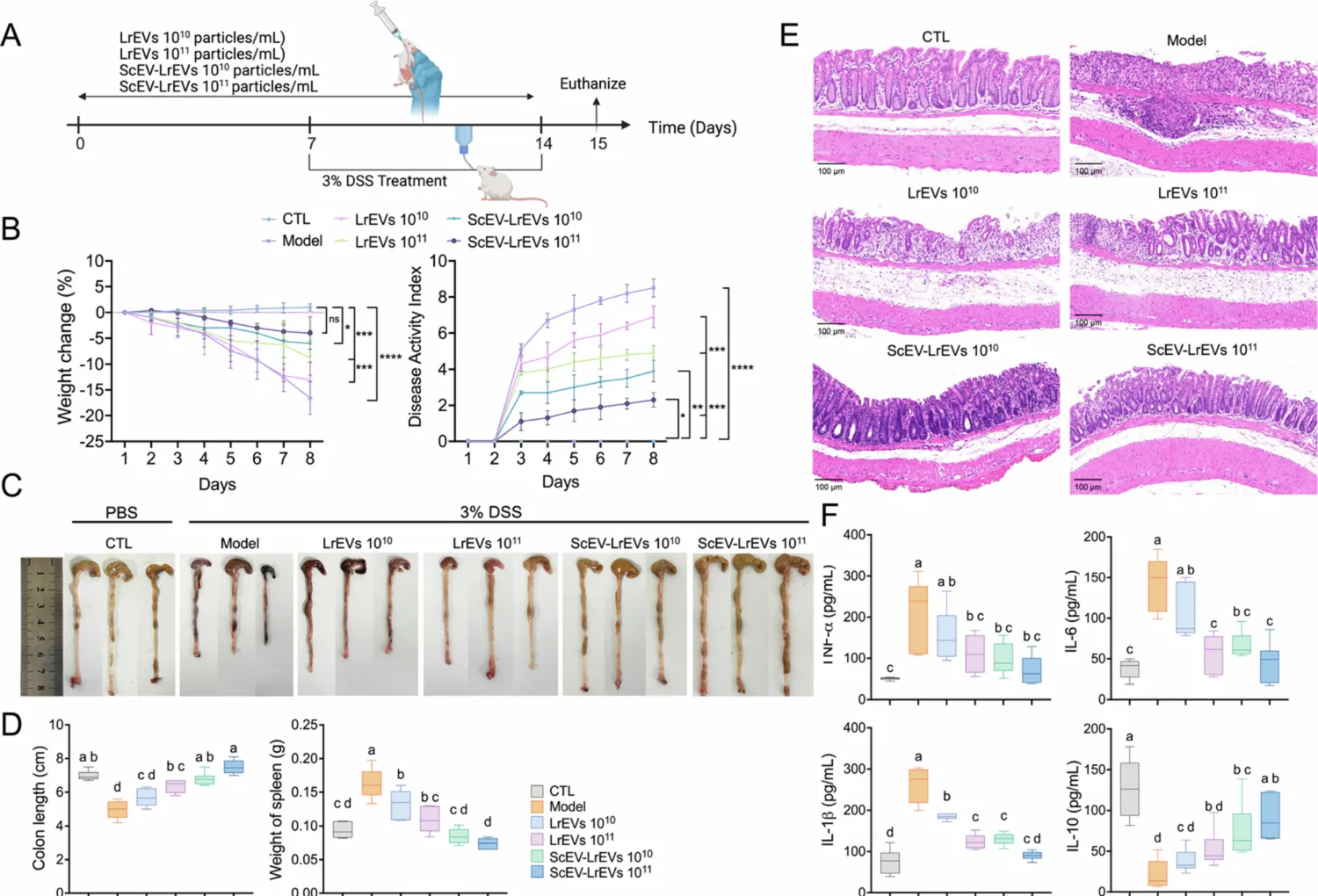
Protective effects of LrEVs and ScEV-LrEVs against DSS-induced colitis in mice. (A) Experimental timeline showing daily oral administration of PBS, LrEVs, or ScEV-LrEVs from day 1 to day 15 and induction of colitis with 3% DSS from day 8 to day 14 (n = 6 mice per group). (B) Changes in body weight and disease activity index (DAI) throughout the experimental period (n = 6 per group). (C−D) Representative colon morphology, colon length, and spleen weight (n = 6 per group). (E) H&E staining of colon sections (scale bar, 100 μm, representative sections from n = 6 mice per group). (F) Serum levels of cytokines (TNF-α, IL-6, IL-1β, and IL-10) determined by ELIZA (n = 6 per group). Quantitative data are presented as mean ± SD. Body weight and DAI were analyzed using two-way repeated-measures ANOVA followed by Tukey’s multiple-comparisons test. Terminal outcomes were analyzed using one-way ANOVA followed by Tukey’s multiple-comparisons test.

To evaluate the therapeutic potential of ScEV-conditioned *Lactobacillus* vesicles *in vivo*, a 3% DSS-induced colitis model was established in mice (Figure 4A). Mice received oral LrEVs or ScEV-LrEVs at doses of 10^10^ or 10^11^ particles/mL throughout the 15-day experimental period, with DSS administered in the drinking water from day 8 to day 14. DSS exposure induced progressive body weight loss and high disease activity index (DAI), both significantly attenuated by EV treatment, with the greater protection observed in ScEV-LrEVs-treated mice than the LrEV group (Figure 4B). Macroscopic examination revealed severe colonic shortening, congestion, and edema in DSS-treated mice, which were substantially alleviated by EV administration (Figure 4C). ScEV-LrEVs restored colon length and reduced spleen hypertrophy to near-normal levels, showing a stronger protective effect than LrEVs treatment (Figure 4D). H&E staining showed that DSS exposure caused extensive mucosal erosion, crypt distortion, goblet cell depletion, and dense leukocyte infiltration (Figure 4E). In contrast, mice treated with ScEV-LrEVs, especially at higher doses, retained epithelial continuity and crypt organization with reduced inflammatory infiltration.

**Figure 5.**
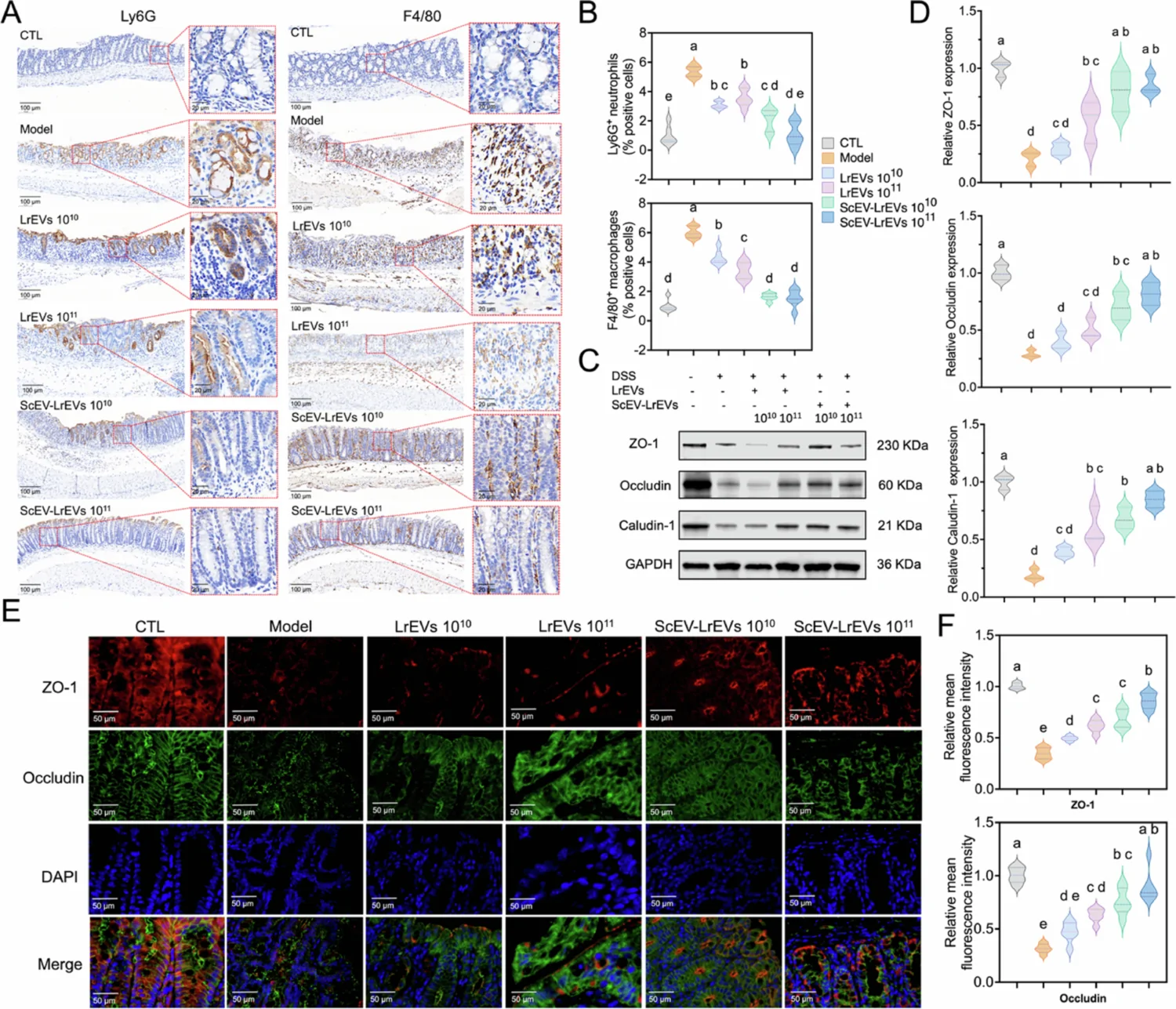
ScEV-LrEVs alleviate colonic inflammation and restore intestinal barrier integrity in DSS-induced colitis. (A) Immunohistochemical staining of colon tissue showing neutrophil (Ly6G+) and macrophage (F4/80+) infiltration (scale bar, 100 μm, representative sections from n = 6 mice per group). (B) Quantification of Ly6G+ and F4/80+ cells (n = 6 per group). (C–D) Western blot images and quantification of tight junction proteins ZO-1, Occludin, and Claudin-1 (n = 6 per group). (E) Immunofluorescence staining of colon sections showing ZO-1 (red) and Occludin (green) localization along the epithelial junctions (DAPI, blue; scale bar, 50 μm, representative sections from n = 6 mice per group). (F) Quantification of ZO-1 and Occludin fluorescence intensity (n = 6 per group). Quantitative data are presented as mean ± SD. Group differences were analyzed using one-way ANOVA followed by Tukey’s multiple-comparisons test.

Serum cytokine analysis showed lower levels of TNF-*α*, IL-6, and IL-1β and higher levels of IL-10 in EV-treated mice compared with DSS controls (Figure 4F). Notably, the ScEV-LrEVs group exhibited stronger, dose-dependent anti-inflammatory efficacy than unconditioned LrEVs. Immunohistochemical staining showed that DSS markedly increased Ly6G^+^ neutrophil and F4/80^+^ macrophage infiltration, while EV administration, particularly ScEV-LrEVs, significantly reduced their abundance (Figure 5A and B).

To assess epithelial barrier function, we examined the tight junction proteins ZO-1, occludin, and claudin-1, which are essential for maintaining intestinal permeability (Figure 5C). DSS treatment markedly decreased their expression, as confirmed by western blot and immunofluorescence analyzes (Figures 5C−F). ScEV-LrEVs substantially restored their expression and proper localization along the epithelial border. Overall, these results demonstrate that ScEV-conditioning enhances the anti-inflammatory effect of *Lactobacillus* vesicles in DSS-induced colitis.

### 2.5. ScEV-conditioned *Lactobacillus* vesicles are associated with restoration of lysosomal function in colonic tissues

**Figure 6.**
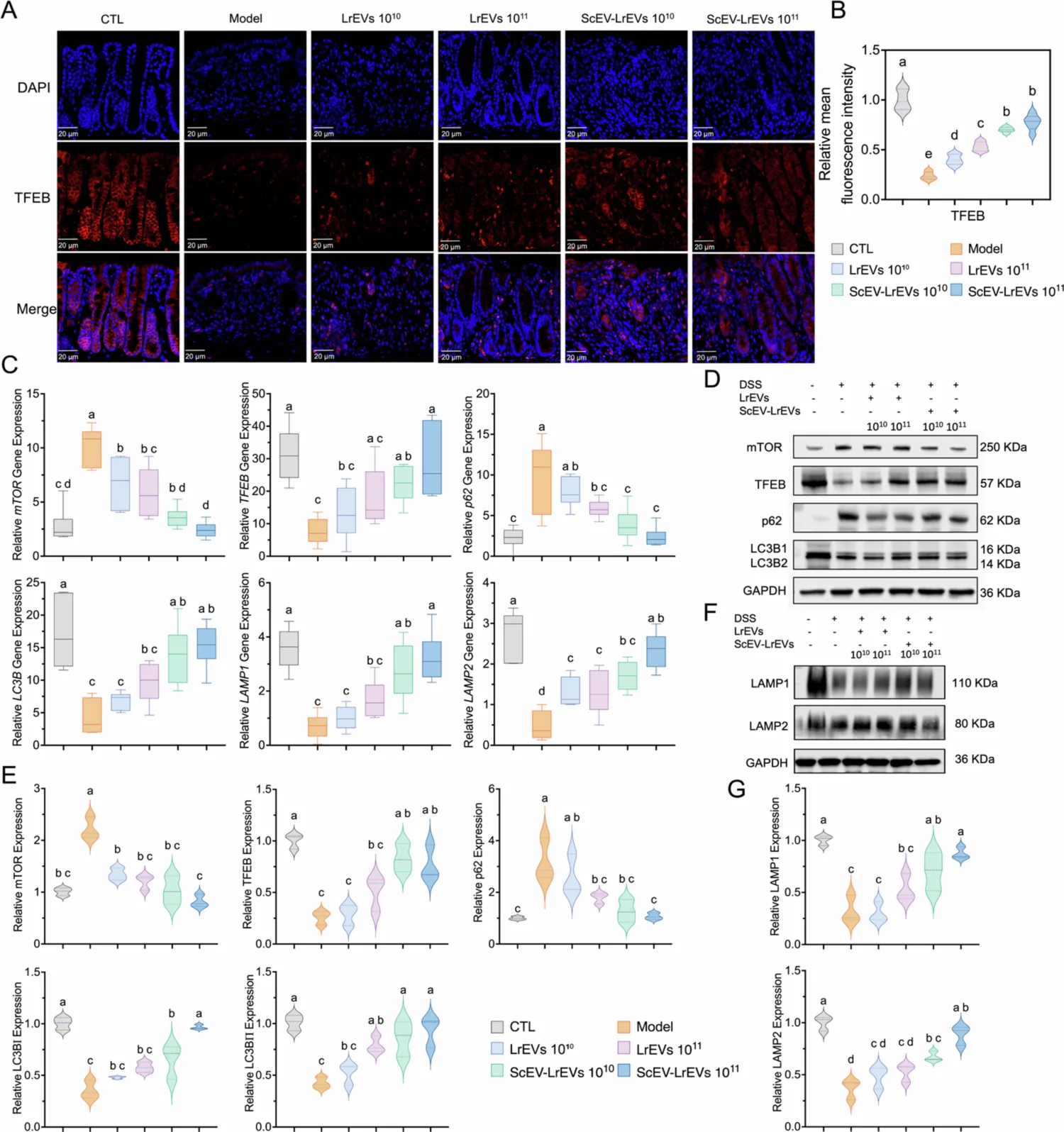
ScEV-LrEVs modulate the mTOR-TFEB-lysosome pathway in DSS-induced colitis. (A) Immunofluorescence of TFEB (red) and nuclei (blue) in colon tissues (scale bar, 20 μm, representative sections from n = 6 mice per group). (B) Quantification of TFEB intensity (n = 6 per group). (C) Relative mRNA expression levels of mTOR-TFEB pathway genes (mTOR, TFEB, p62, LC3B, LAMP1, and LAMP2) (n = 6 per group). (D−E) Western blot images and quantification of mTOR, TFEB, p62, and LC3B-I/II (n = 6 per group). (F−G) Western blot images and quantification of LAMP1 and LAMP2 (n = 6 per group). Quantitative data are presented as mean ± SD. Group differences were analyzed using one-way ANOVA followed by Tukey’s multiple-comparisons test.

To determine whether the lysosome- and autophagy-associated molecular changes observed in macrophages were also present *in vivo*, we examined lysosomal and mTOR-TFEB-related markers in colonic tissues from DSS-treated mice administered with LrEVs or ScEV-LrEVs. Immunofluorescence (Figure 6A−B) and immunohistochemical staining (Fig. S6A-B) consistently showed that DSS exposure markedly reduced TFEB signal intensity along the epithelial layer, indicative of impaired lysosomal activity under inflammatory conditions. Treatment with LrEVs partially restored TFEB expression, whereas ScEV-LrEVs induced a more pronounced recovery, suggesting a stronger restoration of TFEB-associated lysosomal responses *in vivo*.

Western blot and qRT-PCR analyzes further substantiated these findings, showing consistent molecular alterations at both the transcript and protein levels. DSS treatment markedly increased *mTOR* and *p62* expression while suppressing *TFEB*, *LC3B*, *LAMP1*, and *LAMP2* (Figure 6C−G), indicative of impaired autophagy and lysosomal function. Both EV treatments mitigated these changes, with ScEV-LrEVs exerting a more pronounced restorative effect than native LrEVs across both molecular layers.

Overall, these findings mirror the cellular results and indicate that ScEV conditioning enhances the ability of *Lactobacillus* EVs to normalize TFEB-related lysosomal and autophagy-associated markers in colonic tissues. These molecular changes were accompanied by improved mucosal protection in DSS-induced colitis.

### 2.6. ScEV-conditioned *Lactobacillus* vesicles exhibit proteomic and lipidomic remodeling associated with anti-inflammatory activity

To uncover the molecular basis of the enhanced anti-inflammatory effects of ScEV-conditioned vesicles, we performed comparative proteomic and lipidomic analyzes of LrEVs and ScEV-LrEVs. Quantitative proteomics showed that ScEV exposure reduced the total number of proteins and peptides (Fig. S7A), indicating a more streamlined vesicular proteome. Principal component analysis (PCA) revealed clear group separation (Fig. S7B), with 30 proteins upregulated and 69 downregulated in ScEV-LrEVs (Fig. S7C). Functional enrichment analyzes indicated that these proteins were mainly associated with lipid and carbohydrate metabolism, proteolysis, and membrane transport (Figs. S7D-E). KEGG pathway mapping further highlighted fatty acid and glycerophospholipid metabolism, as well as glycosphingolipid biosynthesis, suggesting that ScEVs reshape bacterial vesicle formation through metabolic and membrane-associated processes.

**Figure 7.**
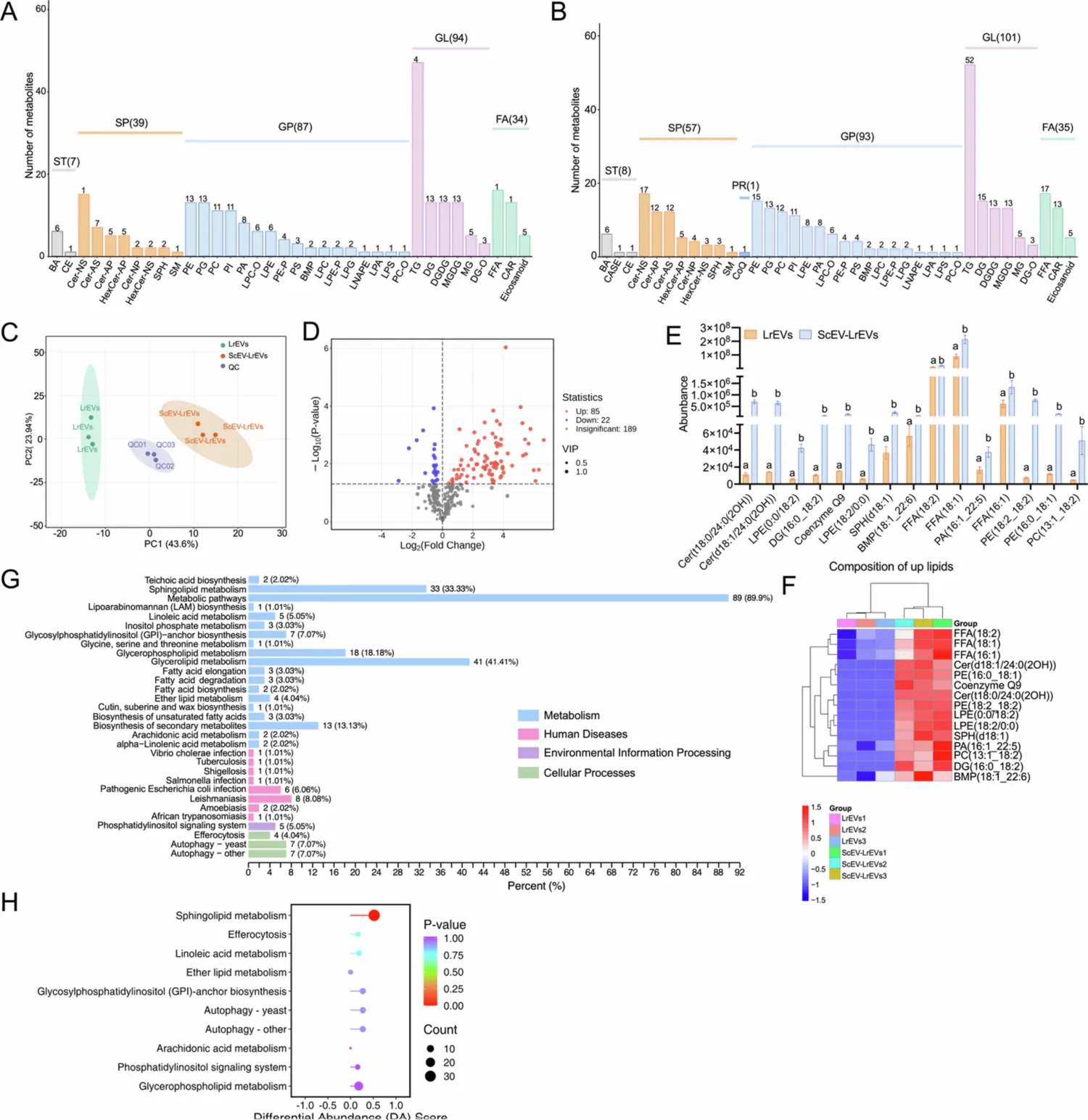
ScEV-conditioning remodels the lipid composition of Lactobacillus EVs. (A) Lipid class distribution of native LrEVs (n = 3 independent EV preparations per group). (B) Lipid class distribution of ScEV-LrEVs after co-culture with ScEVs (n = 3 independent EV preparations per group). (C) Principal component analysis (PCA) revealing clear separation between LrEVs and ScEV-LrEVs, indicating distinct lipidomic signatures (n = 3 independent EV preparations per group). (D) Volcano plot of differentially abundant lipid species between LrEVs and ScEV-LrEVs (VIP > 0.5, p < 0.05), (n = 3 independent EV preparations per group). (E) Top 15 upregulated lipids in ScEV-LrEVs compared with LrEVs (n = 3 independent EV preparations per group). (F) Hierarchical clustering heatmap of the top 15 upregulated lipid species, illustrating consistent increases across biological replicates (n = 3 independent experiments). (G) KEGG pathway enrichment analysis (n = 3 independent EV preparations per group). (H) Bubble plot of significantly enriched pathways, emphasizing changes in sphingolipid metabolism, glycerophospholipid pathways, phosphatidylinositol signaling, autophagy-related pathways, and inflammatory regulation (n = 3 independent EV preparations per group). Quantitative data are presented as mean ± SD, and statistical analyzes were performed as described in the main methods.

Lipidomic profiling revealed even greater changes (Figure 7). ScEV-LrEVs exhibited expanded sphingolipid (SP) and glycerophospholipid (GP) classes, including newly detected coenzyme Q-related species (Figure 7A−B). PCA confirmed distinct clustering (Figure 7C), while volcano plot analysis identified 85 upregulated and 22 downregulated lipid species (Figure 7D). Notably, relative lipidomic analysis showed that hydroxylated ceramides, including Cer(t18:0/24:0(2OH)) and Cer(d18:1/24:0(2OH)), were enriched in ScEV-LrEVs by approximately 60-fold and 40-fold, respectively (Figure 7E). These bioactive lipids are known regulators of vesicle dynamics and lysosomal autophagy and have been associated with TFEB-mediated transcription.^18^ Additional increases in free fatty acids (16:1) and phospholipids such as PE (18:2/18:2) and PC (13:1/18:6) reflected enhanced membrane remodeling and inflammation resolution,^19^ while triacylglycerols and long-chain diacylglycerols were significantly reduced (Figs. S8A and B), indicating a shift from storage to structural and signaling lipids.

KEGG enrichment of differential lipids emphasized sphingolipid and glycerophospholipid metabolism, linoleic acid metabolism, and phosphatidylinositol signaling (Figure 7G), pathways closely related to vesicle secretion and autophagy (Figure 7H). Sphingolipids and glycerophospholipids regulate membrane curvature and fusion critical for vesiculation, while phosphatidylinositol- and linoleic acid-linked pathways are recognized modulators of the mTOR-TFEB axis.^20^ Together, these multi-omics data indicate that ScEV conditioning is associated with marked remodeling of the protein and lipid architecture of *Lactobacillus*-derived EVs, including enrichment of bioactive sphingolipids and phospholipids potentially linked to lysosome-autophagy regulation and inflammation resolution.

### 2.7. Cer(t18:0/24:0(2OH)) reconstitution partially recapitulates the lysosome-restorative and anti-inflammatory activity of ScEV-LrEVs

**Figure 8.**
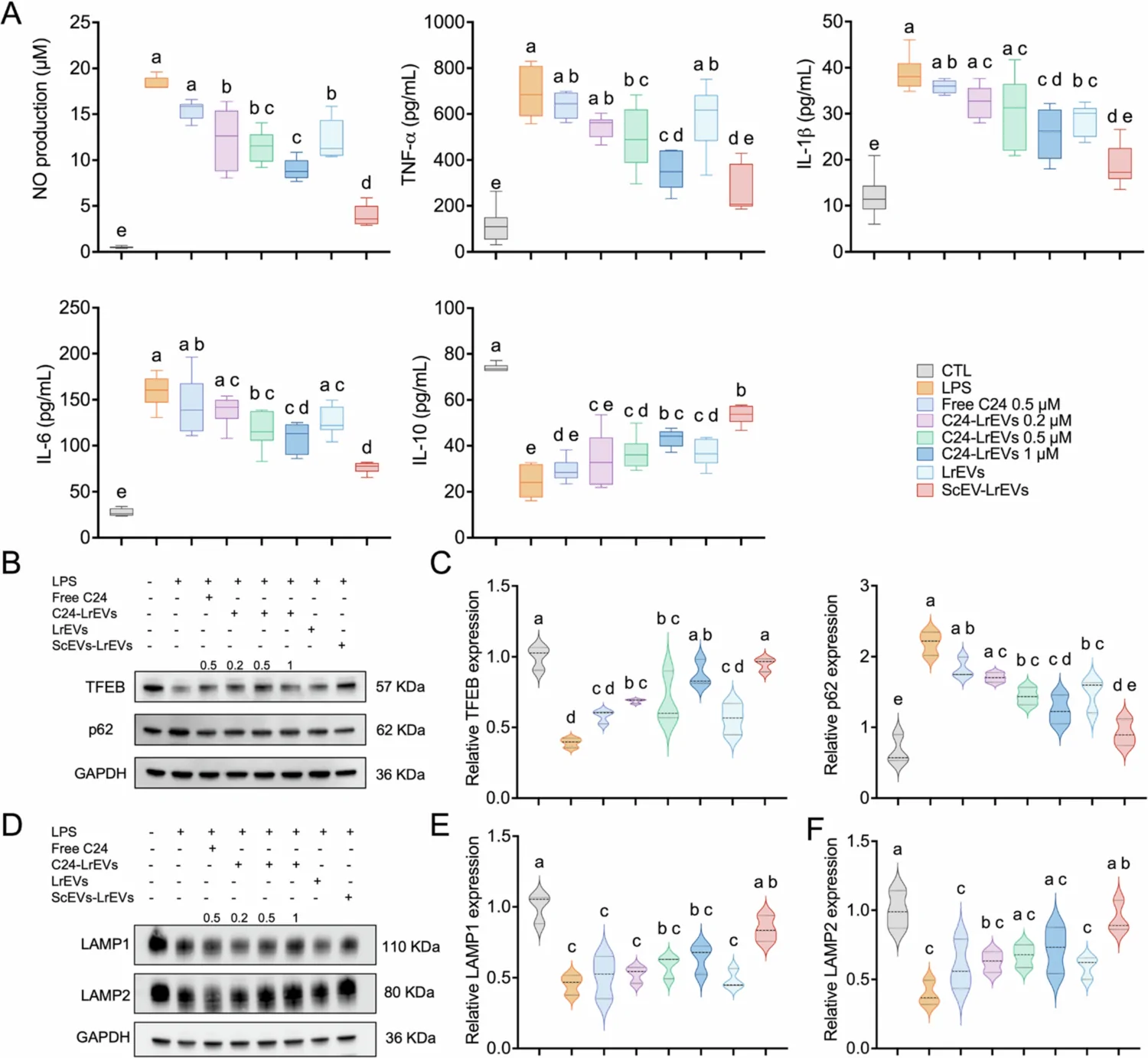
Cer(t18:0/24:0(2OH)) recapitulates the enhanced anti-inflammatory activity of ScEV-LrEVs. (A) Nitric oxide levels in LPS-stimulated macrophages after treatment with free ceramide (0.5 μM), ceramide-loaded LrEVs (0.2, 0.5, or 1 μM), native LrEVs, or ScEV-LrEVs (n = 3 independent experiments). (B) Cytokine levels of TNF-α, IL-1β, IL-6, and IL-10 after the indicated treatments, determined by ELIZA (n = 3 independent experiments). (C–D) Representative Western blot images and quantification of TFEB and p62 (n = 3 independent experiments). (E–F) Western blot images and quantification of LAMP1 and LAMP2 (n = 3 independent experiments). Quantitative data are presented as mean ± SD, and statistical analyzes were performed as described in the main methods.

Building on lipidomic evidence of hydroxylated ceramide enrichment in ScEV-conditioned vesicles, we investigated whether the most prominently enriched ceramide species could functionally contribute to the enhanced anti-inflammatory activity. Among them, Cer(t18:0/24:0(2OH)) (C24) was the most prominently upregulated species identified in ScEV-LrEVs. The dose range for C24 reconstitution was selected based on preliminary cytotoxicity screening and EV-loading feasibility. Cytotoxicity analysis showed that free C24 exhibited dose-dependent toxicity above 2.5 μM (Fig. S9A), whereas C24-loaded LrEVs (C24-LrEVs) were well tolerated up to 5 μM (Fig. S9B). Therefore, subsequent functional assays were performed within the non-cytotoxic range of C24-LrEVs. In LPS-stimulated RAW264.7 macrophages, free C24 modestly suppressed inflammatory mediators, while C24-LrEVs significantly reduced nitric oxide production and the secretion of TNF-*α*, IL-1β, and IL-6, accompanied by elevated IL-10 release (Figure 8A−B). These effects were dose-dependent, reaching maximal efficacy at 1 μM C24 loading. Although C24-LrEVs did not fully match the potency of ScEV-LrEVs, they consistently outperformed native LrEVs, indicating that hydroxylated ceramide incorporation can partially confer enhanced anti-inflammatory activity to LrEVs.

Immunoblot analysis further revealed that C24-LrEVs restored lysosomal-autophagy signaling disrupted by LPS stimulation. Treatment reversed TFEB suppression and p62 accumulation in a concentration-dependent manner (Figure 8C−D) and upregulated lysosomal membrane proteins LAMP1 and LAMP2 (Figure 8E−F), closely resembling the effects of ScEV-LrEVs. These results support Cer(t18:0/24:0(2OH)) as an important contributory lipid that can reinforce lysosomal homeostasis and support anti-inflammatory signaling when incorporated into LrEVs.

Collectively, these data show that ScEV conditioning generates *L. rhamnosus* EVs with altered molecular composition and enhanced anti-inflammatory activity under the defined *in vitro* conditioning system, and that these isolated ScEV-LrEVs retain protective activity when administered *in vivo*. This lipid remodeling was accompanied by enhanced lysosomal function, consistent with the improved anti-inflammatory responses observed. Functional reconstitution with Cer(t18:0/24:0(2OH)) partially recapitulated key lysosome-restorative and anti-inflammatory phenotypes, suggesting a contributory role for sphingolipid remodeling in this cross-kingdom vesicular communication and host immune regulation.

## 3. Discussion

Extracellular vesicles (EVs) have emerged as critical mediators of host microbiome communication that influence immune balance,^21^ epithelial integrity,^22^ and metabolic signaling.^23^ Although bacterial EVs are often examined for their direct effects on host cells, growing evidence indicates that exogenous EVs, particularly those from plants and fungi, can also enter the gastrointestinal tract and interact with resident microbes, thereby influencing microbial metabolism and systemic physiology.^14^ For example, garlic-derived exosome-like nanoparticles have been shown to condition gut bacteria and alter the functional properties of their subsequently released outer membrane vesicles, thereby improving metabolic outcomes.^24^ In addition, our previous study demonstrated that *Lentinula edodes*-derived exosome-like nanoparticles interact with the gut microbiota and enhance the stability and microbial biotransformation of curcumin, further supporting the capacity of edible fungal vesicles to regulate microbiota-associated metabolic processes.^25^ These observations highlight the possibility of cross-kingdom vesicular modulation within the gut ecosystem. However, whether and how plant- or fungus-derived EVs modulate microbial metabolism and remodel the molecular and functional properties of microbiota-derived EVs remains largely unexplored.

In the present study, we identified a cross-kingdom vesicle interaction in which EVs derived from the edible fungus *Schizophyllum commune* were associated with selected gut bacterial populations and altered the abundance and cargo composition of EVs recovered from *Lactobacillus* cultures. ScEV conditioning increased the net EV output of *L. rhamnosus* cultures and generated ScEV-conditioned vesicles with enhanced anti-inflammatory activity. Compared with native LrEVs, ScEV-LrEVs showed more pronounced effects in LPS-stimulated macrophages and DSS-induced colitis, accompanied by improved lysosomal function and coordinated changes in TFEB-, autophagy-, and lysosome-related markers. Comparative proteomic and lipidomic analyzes further revealed extensive remodeling of ScEV-LrEV cargo composition, including marked enrichment of hydroxylated ceramides. These findings collectively support a model in which fungal EV exposure remodels the molecular and functional properties of bacterial EVs and enhances their host-protective activity. The responsiveness to ScEVs may not be uniform across *Lactobacillus species* or strains. Differences in bacterial cell-envelope architecture, including surface-associated proteins, peptidoglycan organization, teichoic acids, extracellular polysaccharides, and membrane lipid composition, may influence vesicle-bacterium association and subsequent membrane interactions or cargo transfer.^26^ ^,^ ^27^ Such strain-dependent surface and membrane properties could therefore contribute to the preferential association of ScEVs with particular bacterial populations. However, the molecular determinants governing ScEV-bacterium interactions were not directly examined in the present study. Because the detailed conditioning and downstream mechanistic analyzes were primarily performed using *L. rhamnosus*, whether the observed response is conserved across other *Lactobacillus species* or represents a strain-dependent phenomenon remains to be determined.

To investigate how exogenous fungal EV exposure influences subsequently released bacterial EVs, we established a ScEV-conditioning system in which *L. rhamnosus* was exposed to ScEVs for 6 h, followed by washing and further culture in fresh medium before bacterial EV isolation. This experimental design was intended to minimize ScEV carryover and to evaluate the vesicles subsequently produced by ScEV-conditioned bacteria. Notably, bacterial EV production and cargo composition are sensitive to environmental conditions, supporting the view that vesicle-mediated bacterial responses are dynamic and environmentally regulated.^28^ Interactions between exogenous vesicles and bacteria may involve several concurrent processes, including vesicle association or uptake, cargo utilization, bacterial adaptation, and subsequent EV secretion. Therefore, the measured EV yield represents the net vesicle output after ScEV conditioning rather than a time-resolved assessment of ScEV uptake, degradation, consumption, and newly induced bacterial EV secretion during the initial interaction period. Moreover, because ScEV exposure also promoted bacterial growth and EV yield was not normalized to bacterial biomass or viable cell number, the present data cannot distinguish whether the increased EV output resulted from greater bacterial abundance, enhanced vesicle release on a per-cell basis, or a combination of both. Thus, the current findings do not establish a direct effect of ScEVs on the intrinsic vesiculation or EV biogenesis capacity of *L. rhamnosus*. Future studies combining biomass-normalized EV quantification with analyzes of bacterial membrane remodeling and EV biogenesis will be required to distinguish these possibilities.

Having established ScEV-conditioned bacterial vesicles, we next considered whether this remodeling of bacterial EVs translated into functional alterations. Compared with native LrEVs, ScEV-LrEVs markedly reduced nitric oxide and the pro-inflammatory cytokines TNF-*α*, IL-6, and IL-1β while increasing IL-10 production in LPS-stimulated macrophages. These anti-inflammatory effects were accompanied by increased LysoTracker fluorescence, elevated LAMP1 and LAMP2 expression, and reduced p62 accumulation, consistent with improved lysosomal function and lysosome-autophagy-associated responses. The attenuation of these effects by chloroquine further supports the contribution of intact lysosomal function to the activity of ScEV-LrEVs. Previous studies have similarly shown that bacterial EVs can regulate host autophagy and inflammatory responses. For instance, *E. coli* EVs activate autophagy through TLR4-NF-κB signaling,^29^ and *Lactobacillus plantarum*-derived EVs improve colitis outcomes by restoring lysosomal degradation.^30^ In the present study, ScEV-LrEVs also alleviated DSS-induced colitis, reduced inflammatory-cell infiltration, preserved epithelial structure, and restored tight-junction protein expression. These observations are consistent with previous reports that gut microbial EVs regulate mucosal immunity, reinforce epithelial barrier integrity, and influence macrophage polarization.^31^ ^,^ ^32^

The enhanced anti-inflammatory activity of ScEV-LrEVs was consistently accompanied by improved lysosomal function, suggesting that lysosomal homeostasis may represent a key mechanistic link underlying their biological effects. Lysosomes are increasingly recognized as regulators of inflammatory resolution rather than merely terminal degradative organelles. Impaired lysosomal acidification or defective autophagosome–lysosome fusion can lead to the accumulation of damaged organelles and protein aggregates, thereby promoting oxidative stress and sustaining inflammatory signaling.^33^ ^,^ ^34^ EVs may both regulate and be processed by autophagy-lysosome pathways, forming a reciprocal relationship between vesicular communication and cellular clearance.^35^ Bacterial EVs have also been reported to engage host autophagic machinery and modulate immune homeostasis.^36^ In this context, the improved lysosomal activity and coordinated changes in TFEB, p62, LC3B, LAMP1, and LAMP2 observed following ScEV-LrEV treatment suggest that restoration of lysosomal homeostasis may contribute to its enhanced anti-inflammatory activity. Together, these findings support restoration of lysosomal homeostasis as a central mechanism associated with the enhanced activity of ScEV-LrEVs, while the causal requirement of the upstream mTOR-TFEB axis remains to be established.

To better understand the molecular basis underlying the enhanced biological activity of ScEV-LrEVs, we next considered whether ScEV conditioning altered the molecular composition of bacterial EVs, with particular attention to their lipid composition. Lipidomic profiling showed that ScEV-LrEVs possessed a distinct lipid composition characterized by broad remodeling of sphingolipids and glycerophospholipids. In addition to the marked enrichment of hydroxylated ceramides, increased levels of free fatty acid 16:1 and phospholipids, including PE(18:2/18:2) and PC(13:1/18:6), were observed, whereas several triacylglycerols and long-chain diacylglycerols were reduced. Sphingolipids and glycerophospholipids are important structural and signaling components of vesicle membranes and may influence membrane curvature, vesicle formation, fusion, and intracellular trafficking.^37^ Changes in free fatty acids and phospholipids may also affect inflammatory signaling and membrane-associated cellular responses.^38^ Together, these findings indicate that ScEV conditioning induces coordinated lipid remodeling rather than alteration of a single lipid species.

Among the upregulated lipids, the hydroxylated ceramides Cer(t18:0/24:0(2OH)) and Cer(d18:1/24:0(2OH)) showed particularly pronounced enrichment, with Cer(t18:0/24:0(2OH)) exhibiting the greatest increase. Hydroxylated ceramides have been associated with membrane organization, lysosomal stability, acidification, and autophagosome-lysosome fusion.^39^ ^,^ ^40^ On this basis, Cer(t18:0/24:0(2OH)) was selected for subsequent functional reconstitution. C24-loaded LrEVs exerted stronger anti-inflammatory and lysosome-restorative effects than native LrEVs, although their activity did not fully reach that of ScEV-LrEVs. These findings indicate that C24 enrichment contributes to, but is unlikely to fully account for, the enhanced activity of ScEV-conditioned vesicles. The broader proteomic and lipidomic remodeling observed in ScEV-LrEVs suggests that multiple cargo components may act cooperatively to regulate lysosomal homeostasis and inflammatory responses. Notably, C24 reconstitution was also accompanied by concentration-dependent reversal of TFEB suppression and p62 accumulation, together with increased LAMP1/2 levels. However, these findings do not establish that C24 directly regulates the mTOR-TFEB pathway. C24 may instead influence lysosomal membrane organization or other lipid-dependent processes that improve lysosomal homeostasis, with the observed TFEB-related changes occurring downstream of these effects. Alternatively, both lysosomal recovery and TFEB normalization may reflect a broader restoration of cellular homeostasis. Thus, the precise molecular sequence linking EV-associated C24 to TFEB-related signaling remains to be determined.

Other prominently altered lipid species may also contribute to the lysosome-restorative effects of ScEV-LrEVs. In particular, bis(monoacylglycero)phosphate (BMP), which is enriched in late endosomal and lysosomal membranes, plays important roles in lysosomal lipid catabolism, lipid hydrolase activation, and membrane-associated lipid processing.^41^ Sphingosine-related lipids may likewise contribute to lysosomal and immune regulatory processes. Thus, the selection of C24 for functional reconstitution was based on its pronounced enrichment and biological relevance and was intended as a representative gain-of-function test, rather than as evidence that C24 is the sole lipid mediator. The incomplete phenocopy observed with C24-LrEVs further supports the possibility that BMP, sphingosine-related lipids, and other remodeled lipid species may act cooperatively in regulating lysosomal homeostasis and inflammatory responses. Rather than acting through a single dominant lipid species, the enhanced activity of ScEV-LrEVs may therefore reflect coordinated lipid remodeling, in which different lipid classes provide complementary structural and signaling contributions. Hydroxylated ceramides may contribute to membrane organization and lysosomal homeostasis, whereas BMP and other phospholipids may support lysosomal membrane-associated lipid processing, with sphingosine-related lipids potentially providing additional regulatory inputs. This multi-lipid model is consistent with the partial, rather than complete, phenotypic recapitulation achieved by C24 reconstitution and remains to be functionally dissected in future studies.

An important question arising from these findings is the origin of the enriched hydroxylated ceramides. Unlike well-characterized sphingolipid-producing gut bacteria such as *Bacteroides*, *Lactobacillus* species are not generally regarded as major de novo sphingolipid producers.^42^ ^,^ ^43^ Two non-mutually exclusive hypotheses may therefore account for the altered lipid composition of ScEV-LrEVs. First, fungal lipids or lipid intermediates associated with ScEVs may be transferred to *L. rhamnosus* and subsequently incorporated into bacterial EVs. Second, ScEV-derived signals or cargo may induce bacterial metabolic and membrane remodeling, thereby altering the lipid composition of subsequently released EVs. These two processes may also occur concurrently. Although bacteria were washed before re-culture and EV isolation, this procedure minimizes free ScEV carryover but cannot completely exclude trace transfer of ScEV-derived lipids or other fungal components. Therefore, the present lipidomic data cannot distinguish fungal lipid transfer from ScEV-induced bacterial lipid reprogramming, and the observed lipid differences should not be attributed solely to endogenous bacterial metabolic remodeling. Species-specific vesicle markers, isotope tracing, or dual-labeling approaches will be required to distinguish these possibilities.

Beyond the molecular remodeling of bacterial EVs, our findings also distinguish this mechanism from previously reported dietary EV systems. Orally administered dietary EVs may protect intestinal barrier integrity through direct cargo-dependent mechanisms. For example, milk-derived EVs have been reported to reinforce mucus, epithelial, and immune barriers through their endogenous molecular cargo, including miRNAs.^44^ However, the present study was specifically designed to investigate an indirect microbiota-mediated route through which exogenous fungal EVs condition gut bacteria and alter the properties of subsequently released bacterial EVs. The host-cell-free conditioning system supports a direct interaction between ScEVs and *L. rhamnosus* during the generation of ScEV-LrEVs. Nevertheless, ScEVs may also act directly on intestinal epithelial or immune cells after administration. Because the DSS colitis experiment compared LrEVs with ScEV-LrEVs but did not include an ScEV-alone group, the present *in vivo* design cannot distinguish the contribution of direct ScEV-host interactions from indirect protection mediated through ScEV-conditioned bacterial EVs. These two routes are not mutually exclusive and may operate concurrently *in vivo*. An ScEV-alone group would help establish the overall protective activity of ScEVs, whereas quantitative separation of direct host-cell effects from microbiota-mediated effects will require more systematic experimental designs incorporating ScEV administration with microbiota manipulation and complementary host-cell or microbiota-host models. Importantly, the ScEV-LrEVs used for the cellular and animal functional experiments in this study were generated and isolated using the defined *in vitro* conditioning system and were subsequently administered at controlled particle concentrations. Thus, these experiments establish the biological activity of experimentally generated ScEV-LrEVs, but do not demonstrate that oral administration of ScEVs produces equivalent quantities of functional ScEV-conditioned *Lactobacillus* EVs in the intestinal environment. The short-term bacterial-association experiments and the increased relative abundance of *Lactobacillaceae* following long-term ScEV administration support *in vivo* contact between ScEVs and *Lactobacillus*-related populations, but they do not establish the identity, abundance, or functional activity of bacterial EVs generated as a consequence of this interaction. In the absence of specific markers distinguishing native *Lactobacillus* EVs from ScEV-conditioned EVs, their production cannot currently be selectively quantified within the complex intestinal vesicle pool. Therefore, the ScEV-*L. rhamnosus*-EV pathway demonstrated in the defined conditioning system should be regarded as a mechanistic model supported by, but not directly demonstrated in, the *in vivo* setting. In addition, whether ScEV-LrEVs further reshape the intestinal microbiota after administration remains to be investigated. Although long-term ScEV administration altered gut microbial composition, microbiota changes following ScEV-LrEV treatment were not evaluated in the DSS model. Microbiota profiling, microbiota depletion, and fecal microbiota transplantation could further clarify whether the protective effects of ScEV-LrEVs involve secondary modulation of the intestinal microbial community.

Several limitations should be considered. First, ScEV biodistribution and gut bacterial association were evaluated in healthy mice, and their intestinal accessibility under inflammatory conditions was not directly examined. Disruption of the intestinal barrier and changes in mucus organization, epithelial permeability, and microbial ecology during intestinal inflammation may alter the retention, distribution, and interactions of orally administered vesicles. Therefore, the biodistribution and bacterial-association patterns observed in healthy mice should not be directly extrapolated to the inflamed intestine, and the effective interaction of ScEVs with gut bacteria under inflammatory conditions remains to be established. Future studies using vesicle tracing in inflammatory models will be required to define the spatial distribution, persistence, and microbiota interactions of ScEVs in the diseased intestine. In addition, because an ScEV-alone group was not included in the DSS colitis experiment, the present *in vivo* data cannot separate potential direct effects of ScEVs on host epithelial or immune cells from indirect microbiota-mediated effects involving ScEV-conditioned bacterial EVs.

In addition, the amount and functional activity of ScEV-conditioned *Lactobacillus* EVs generated *in vivo* after ScEV administration remain unknown. Because specific molecular markers that distinguish native *Lactobacillus* EVs from ScEV-conditioned EVs are currently lacking, these vesicle populations could not be selectively identified or quantified in the complex intestinal environment. Accordingly, the present study does not establish that oral ScEV administration generates sufficient quantities of functional ScEV-LrEVs *in vivo*, and the mechanistic pathway defined using the *in vitro* conditioning system should not be directly extrapolated to the intestinal setting. Second, although lipidomic analysis identified Cer(t18:0/24:0(2OH)) as one of the most prominently enriched lipids, only relative quantification was performed. Consequently, the reconstitution experiment should be interpreted as a gain-of-function assessment of C24 contribution rather than a precise reproduction of its endogenous abundance. Moreover, because C24 was not selectively depleted from ScEV-LrEVs, the present gain-of-function experiment cannot establish that C24 is required for the biological activity of ScEV-LrEVs. Third, although the present findings link ScEV-LrEV activity to restored lysosomal function and coordinated changes in mTOR-TFEB-related markers, direct causal validation of this regulatory axis remains to be established. TFEB knockdown, TFEB inhibition, or pharmacological activation of mTOR will be required to determine whether this regulatory axis is necessary for the anti-inflammatory effects of ScEV-LrEVs. Finally, before clinical translation, scalable production, batch-to-batch consistency, optimal dosing, long-term safety, vesicle stability, and immunogenicity will require systematic evaluation.

In conclusion, our findings reveal an environmentally responsive form of cross-kingdom vesicular communication in which fungal EVs condition gut *Lactobacillus*, remodel bacterial EV cargo, and enhance the anti-inflammatory activity of the resulting vesicles. This functional remodeling was associated with hydroxylated ceramide enrichment, improved lysosomal homeostasis, and protection against intestinal inflammation. The findings expand the current understanding of exogenous EV-microbiota interactions and suggest that microbial vesiculation may represent a modifiable intermediate linking dietary or fungal vesicles to host intestinal responses. Further clarification of vesicle uptake, cargo transfer, bacterial lipid remodeling, and *in vivo* dose relationships will be important for developing reproducible EV-based strategies for intestinal inflammation.

## 4. Conclusion

This study establishes an *in vitro* cross-kingdom vesicle-conditioning model in which fungal extracellular vesicle exposure alters the molecular and functional properties of *Lactobacillus*-derived EVs. Exposure to ScEVs induced extensive remodeling of the bacterial EV proteome and lipidome, particularly through the enrichment of hydroxylated ceramides. These alterations are consistent with two non-mutually exclusive possibilities: direct transfer of ScEV-associated fungal lipids or lipid intermediates, and ScEV-induced metabolic remodeling within recipient bacteria; the present experiments cannot distinguish between these mechanisms. Functionally, ScEV-conditioned *Lactobacillus* vesicles (ScEV-LrEVs) exhibited superior anti-inflammatory efficacy, suppressing TNF-*α*, IL-6, and IL-1β while enhancing IL-10 production both *in vitro* and *in vivo*. In the DSS-induced colitis model, ScEV-LrEVs alleviated mucosal injury and restored epithelial integrity, with these protective effects being associated with improved lysosomal homeostasis and coordinated changes in mTOR-TFEB-related molecular markers. Collectively, these findings suggest that fungal EV exposure can alter microbial vesicle cargo composition through cross-kingdom interactions involving lipid transfer and/or bacterial metabolic responses, thereby contributing to host immune homeostasis. This work expands the current understanding of microbial cooperation, demonstrating that adaptive remodeling among gut microorganisms can occur through vesicle-driven biochemical exchange. Future studies should identify the key lipid and protein mediators of this cross-microbial communication and determine how dietary or environmental factors modulate these vesicle-based interactions within the gut ecosystem.

## 5. Materials and methods

### 5.1. Preparation of *Schizophyllum commune*-derived extracellular vesicles (ScEVs)

*Schizophyllum commune* was cultured by submerged fermentation in a young apple-based medium, which served both as a carbon source and a model for fruit residue bioconversion, as previously optimized in our laboratory. After 8 days of incubation at 28 °C under shaking conditions, fungal mycelial pellets were harvested. The fermentation broth was homogenized using a sterile high-speed blender to release secreted vesicles into the supernatant. The homogenate was subjected to sequential centrifugation steps to remove cells and large debris (1,000 × g for 10 min, 2,000 × g for 20 min, 4,000 × g for 30 min, 8,000 × g for 60 min, and 15,000 × g for 2 h, all at 4 °C). Supernatants from each step were collected, and the final supernatant was ultracentrifuged at 150,000 × g for 2 h to pellet nanosized vesicles. The resulting pellet was gently resuspended in 1 mL of cold PBS and further purified using sucrose density gradient ultracentrifugation (8%, 30%, 45%, and 60% sucrose in 20 mM Tris-HCl, pH 7.2) at 150,000 × g for 2 h at 4 °C. Purified ScEVs were washed with PBS and characterized morphologically and physicochemically. Transmission electron microscopy (TEM; JEOL, Japan) confirmed their typical cup-shaped morphology, while nanoparticle tracking analysis (NTA; ZetaView PMX 110, Particle Metrix, Germany), dynamic light scattering (DLS; Malvern Zetasizer Nano ZS), and zeta potential measurements determined particle size distribution and surface charge.^45^ For NTA and DLS analysis, three independent ScEV preparations were analyzed, and quantitative particle size and concentration values are reported as mean ± SD from these biological replicates. Representative DLS and NTA profiles are shown in the main figure.

Lipidomic and Proteomic Analyzes of ScEVs. ScEV proteins were lysed in 1% sodium deoxycholate (SDC; Sigma-Aldrich) in 100 mM Tris-HCl (pH 8.5), followed by reduction and alkylation with TCEP (Sigma-Aldrich) and 2-chloroacetamide (CAA; Sigma-Aldrich) at 60 °C for 30 min. After dilution of SDC to below 0.5%, proteins were digested overnight at 37 °C with trypsin at an enzyme-to-protein ratio of 1:50 (w/w; SignalChem). Peptides were analyzed using an UltiMate 3000 RSLCnano system coupled to a Q Exactive HF mass spectrometer (Thermo Fisher Scientific). Raw MS data were processed using MaxQuant version 2.2.0.0 against the *S. commune* UniProt protein database, with a false discovery rate of 1% at both the peptide and protein levels.^46^ For lipidomic profiling, total lipids were extracted from ScEVs using a modified Bligh and Dyer method and analyzed on a Thermo Vanquish UHPLC coupled to a Q Exactive Orbitrap mass spectrometer in both ionization modes. Lipid identification and quantification were performed using LipidSearch (v5.0), and the relative abundance of lipid classes was statistically compared between samples.^47^

### 5.2. *In vivo* biodistribution of ScEVs

General animal information and housing. Eight-week-old male BALB/c and C57BL/6 mice were provided by the Experimental Animal Center of Shaanxi Normal University (Xi’an, China). All mice were maintained under specific pathogen-free conditions in a controlled facility at 22 ± 2 °C and 60 ± 5% relative humidity under a 12-h light/12-h dark cycle, with free access to standard laboratory chow and water unless otherwise specified. All animal procedures were reviewed and approved by the Institutional Animal Care and Use Committee of Shaanxi Normal University (Approval No. 2024-032). Before experimental intervention, mice were allocated to groups using a simple random allocation procedure without stratification or blocking. Individual mice were identified by numbered ear tags, and group allocation remained known to the investigators during treatment, sampling, outcome assessment, and data analysis; therefore, no formal blinding procedure was applied. No formal a priori power calculation was performed. Group sizes were selected based on previously published studies using comparable animal models and the reduction principle of the 3Rs, with the minimum number of animals required to provide adequate biological replication.

To visualize the biodistribution of ScEVs following oral administration, vesicles were labeled with the near-infrared lipophilic dye DiR (MedChemExpress, HY-D1048) according to a previously described procedure with minor modifications.^48^ Briefly, purified ScEVs were incubated with 2 µM DiR at room temperature for 15 min in the dark. To minimize dye-associated artifacts, the labeling reaction was terminated by dilution with sterile PBS, and the suspension was subjected to ultracentrifugation at 150,000 × g for 1.5 h at 4 °C to remove unbound dye. The pellet was gently resuspended in cold sterile PBS and washed by repeated ultracentrifugation for a total of 3 cycles, until the supernatant fluorescence was negligible.

At six predefined terminal time points (0, 2, 6, 12, 24, 48 h), separate cohorts of eight-week-old male BALB/c mice were used, with six mice allocated to each time point (*n* = 6 per time point, 36 mice in total). Each mouse was orally administered 200 µL of DiR-labeled ScEV suspension (1 × 10^10^ particles/mL) using a gavage needle. At the designated time point after administration, mice were anesthetized with isoflurane and subjected to *in vivo* fluorescence imaging using a Bruker Xtreme II *In Vivo* Imaging System (Bruker, Germany). Immediately after *in vivo* imaging, mice were euthanized under deep isoflurane anesthesia, and major organs, including the stomach, small intestine, cecum, colon, liver, spleen, and lung, were collected for ex vivo fluorescence imaging under identical acquisition parameters. Fluorescence intensity in regions of interest was quantified using Bruker Molecular Imaging Software and expressed as total radiant efficiency (photons/s/cm^2^/sr). Statistical analysis was performed using one-way ANOVA followed by Tukey’s post hoc test, with *p* < 0.05 considered statistically significant.

### 5.3. Incubation of ScEVs with human fecal microbiota and bacterial sorting

Fecal Sample Collection and Preparation. The study protocol for human fecal sample collection was reviewed and approved by the Ethics Committee of Shaanxi Normal University (Approval No. 202416036). The study was conducted in accordance with the principles of the Declaration of Helsinki. Written informed consent was obtained from all donors before fecal sample collection. Fresh fecal samples were collected from 10 healthy adult volunteers (5 males and 5 females, aged 25-35 years) who had not received antibiotics or probiotics for at least three months prior to sampling. Using sterile fecal collectors, 2 g of fecal matter from each donor was weighed into sterile 50 mL centrifuge tubes and homogenized with 18 mL of sterile PBS. The suspension was centrifuged at 200 × g for 5 min to remove large debris, and the resulting fecal slurry was mixed with an equal volume of sterile 50% glycerol solution. Aliquots were stored at −80 °C until use.^28^

To examine the interaction between fungal extracellular vesicles and the human gut microbiota, purified ScEVs were fluorescently labeled with PKH26 (MedChemExpress, HY-D1451) according to the manufacturer’s protocol. Briefly, ScEVs were incubated with PKH26 at a final concentration of 1 μM at room temperature for 15 min in the dark with gentle agitation. The labeling reaction was quenched according to the manufacturer’s instructions. Unbound dye was removed by ultracentrifugation at 150,000 × g for 1.5 h at 4 °C. To thoroughly eliminate residual free dye and potential dye aggregates, the pelleted vesicles were resuspended in cold PBS and washed by repeated ultracentrifugation for a total of 3 cycles. Labeled ScEVs were finally resuspended in sterile PBS. Thawed fecal slurries were adjusted to approximately 1 × 10^7^ CFU/mL in anaerobic PBS and incubated with PKH26-labeled ScEVs (1 × 10^10^ particles/mL) at 37 °C for 6 h under anaerobic conditions with gentle agitation. Control samples received equal volumes of PBS. Each experimental group included nine biological replicates, and fecal samples from every three donors were randomly pooled to form one composite replicate (*n* = 3). After incubation, fluorescence microscopy was used to visualize ScEV-associated bacterial cells, confirming successful interaction between vesicles and microbiota. Bacterial cells were collected by centrifugation at 4,000 × g for 10 min, washed three times with cold PBS, and resuspended for fluorescence-activated cell sorting. PKH26-positive bacterial populations were identified and sorted using a MoFlo Astrios flow cytometer (Beckman Colter, USA) under aseptic conditions. Sorted fractions were pelleted and subjected to genomic DNA extraction using the QIAamp DNA Stool Mini Kit (Qiagen, Germany). The V3-V4 region of the 16S rRNA gene was amplified and sequenced on an Illumina NovaSeq platform (Illumina, USA). Raw sequencing reads were processed using QIIME2 for quality control, OTU clustering, and taxonomic classification against the SILVA database. Taxonomic profiles were visualized to illustrate the composition of ScEV-associated bacterial communities.^49^

### 5.4. Validation of ScEV-gut microbiota interactions in mice

Eight-week-old male BALB/c mice were used for the short- and long-term ScEV-gut microbiota interaction studies under the general animal housing and experimental allocation procedures described in Section 5.2. All animal procedures were reviewed and approved by the Institutional Animal Care and Use Committee of Shaanxi Normal University (Approval No. 2024-032).

Short-Term ScEV Tracking and Bacterial Sorting. Purified ScEVs were fluorescently labeled with PKH26 dye (MedChemExpress, HY-D1451) using the labeling and ultracentrifugation-based cleanup procedure described in Section 5.3. The labeled vesicles were resuspended in sterile PBS. The short-term experiment comprised two groups, with nine eight-week-old male BALB/c mice allocated to each group (18 mice in total). Mice were orally administered 200 μL of PKH26-labeled ScEV suspension at a concentration of 1 × 10^10^ particles/mL, corresponding to 2 × 10^9^ particles per mouse, while control animals received an equal volume of PBS. The 3-h sampling time was selected based on preliminary DiR fluorescence imaging showing detectable ScEV-associated signals in the intestinal tract at this early time point. This sampling window was also informed by previous studies showing that orally administered food-derived extracellular nanoparticles can be detected in distal intestinal regions or associated with gut bacterial populations within 3 h after gavage.^24^ At 3 h post-administration, freshly excreted fecal pellets were collected under aseptic conditions, homogenized in sterile PBS, and filtered through a 70 μm nylon mesh. The filtrate was centrifuged at 200 × g for 5 min to remove large debris, followed by centrifugation at 4,000 × g for 10 min. The resulting bacterial pellets were washed twice with PBS. To obtain sufficient sorted bacterial material for downstream sequencing, fecal samples from every three mice within the same treatment group were pooled to generate one composite sample. PKH26-positive bacterial populations were detected and sorted using a MoFlo Astrios flow cytometer (Beckman Colter, USA). The sorted bacterial fractions were collected by centrifugation and subjected to genomic DNA extraction. Thus, the nine mice in each group generated three independent composite sequencing samples per group (*n* = 3 composite samples). The bacterial 16S rRNA gene was amplified and sequenced as described in Section 5.3.^50^

Long-Term Oral Administration and Microbiota Profiling. The long-term experiment comprised two groups, with six mice allocated to each group (12 mice in total). Eight-week-old male BALB/c mice (*n* = 6 per group) were orally administered 200 μL of unlabeled ScEV suspension (1 × 10^10^ particles/mL, corresponding to 2 × 10^9^ particles per mouse) once daily for 30 consecutive days. Control mice received an equal volume of PBS. At the end of the exposure period, animals were anesthetized with isoflurane and euthanized in accordance with ethical standards. Fecal samples were collected and processed for 16S rRNA gene sequencing as described above.

### 5.5. Culture of *Lactobacillu*s strains and preparation of bacterial extracellular vesicles (EVs)

*Lactobacillus rhamnosus* ATCC 53103 and *Lactobacillus reuteri* ATCC PTA-6475 were obtained from the China Center of Industrial Culture Collection (CICC, Beijing, China). Both strains were cultured anaerobically in de Man-Rogosa-Sharpe (MRS) broth at 37 °C using an anaerobic workstation (Whitley DG250, UK). Bacterial growth was determined by OD_600_ measurement.^51^

Co-culture of *Lactobacillus* with ScEVs and Bacterial EV Isolation. To assess the effect of ScEVs on bacterial growth and vesicle production, *L. rhamnosus* and *L. reuteri* were cultured in MRS medium with or without ScEV supplementation (1 × 10^10^ particles/mL). Bacterial suspensions were incubated at 37 °C for 6 h under anaerobic conditions with gentle agitation. After this conditioning period, bacterial cells (together with any cell-associated or internalized ScEVs) were pelleted by low-speed centrifugation (4,000 × g for 5 min), whereas free ScEVs remained in the supernatant, which was discarded. The bacterial pellets were washed three times with sterile PBS to further remove residual unbound ScEVs and then resuspended in fresh MRS medium. The cultures were continued for an additional 48 h to allow extracellular vesicle secretion. This medium-exchange step enabled subsequent EV collection from post-conditioning bacterial cultures, minimizing carryover of free ScEVs from the initial co-incubation. Following incubation, bacterial cells were removed by centrifugation at 10,000 × g for 20 min, and the supernatant was filtered through a 0.22 μm membrane to ensure complete removal of residual cells. The resulting cell-free supernatant was subjected to ultracentrifugation at 150,000 × g for 3 h at 4 °C to pellet bacterial EVs. The EV pellets were gently resuspended in sterile PBS and stored at −80 °C for further analyzes. ScEV-conditioned vesicles were designated as ScEV-LrEVs and ScEV-LreEVs, respectively.^25^ Accordingly, the ScEV-LrEV/ScEV-LreEV preparations were obtained from post-wash, medium-exchanged cultures rather than from the original co-incubation supernatant.

Characterization of Bacterial EVs. The concentration and size distribution of *L. rhamnosus*- and *L. reuteri*-derived EVs were determined using nanoparticle tracking analysis (NTA) following the same procedures described above for ScEV characterization. Particle concentration was expressed as particles per milliliter, and mean diameter was calculated from triplicate measurements.

### 5.6. Cell culture and *in vitro* assays

Cell Culture and Establishment of the Inflammatory Model. Murine macrophage RAW264.7 cells were obtained from Shanghai Fuheng Biotechnology Co., Ltd. (Shanghai, China). Cells were cultured in Dulbecco’s Modified Eagle Medium (DMEM, Gibco, USA) supplemented with 10% fetal bovine serum (FBS, Gibco), 100 U/mL penicillin, and 100 μg/mL streptomycin. Cultures were maintained at 37 °C in a humidified incubator with 5% CO₂. For the inflammatory model, cells were pretreated with EVs at a concentration of 1 × 10^10^ particles/mL for 6 h and were subsequently stimulated with LPS (500 ng/mL; MCE, USA) for an additional 24 h before analysis, following previously described protocols.^17^

Cellular Uptake of Bacterial Extracellular Vesicles. Extracellular vesicles derived from *Lactobacillus rhamnosus* and *Lactobacillus reuteri*, with or without *Schizophyllum commune* conditioning (LrEVs, LreEVs, ScEV-LrEVs, and ScEV-LreEVs), were labeled with PKH26 red fluorescent dye (MCE, USA) according to the manufacturer’s instructions. RAW264.7 cells were incubated with the labeled EVs for 6 h, washed three times with PBS, fixed with 4% paraformaldehyde, and stained with FITC-conjugated wheat germ agglutinin and DAPI (Beyotime, China) for membrane and nuclear visualization, respectively. Fluorescence images were captured using a confocal microscope (Oxford Instruments, UK).^52^

Uptake of ScEVs by Probiotic Strains. To verify the interaction between fungal vesicles and probiotic bacteria, *Lactobacillus rhamnosus* and *Lactobacillus reuteri* were incubated with PKH26-labeled ScEVs (MedChemExpress, HY-D1451) at 37 °C for 6 h under anaerobic conditions. After incubation, bacterial cells were washed, fixed, and visualized using fluorescence microscopy. Red fluorescence signals on bacterial surfaces confirmed the efficient uptake or association of ScEVs by both *Lactobacillus* species.^53^

Cytotoxicity, Nitric Oxide, Cytokine, and ROS Assays. RAW264.7 cells were treated with EVs at different concentrations (10^6^-10^11^ particles/mL) for 24 h. Cell viability was determined using a Cell Counting Kit-8 (CCK-8; Beyotime, China) according to the manufacturer’s protocol. Nitric oxide (NO) production was quantified using a Griess reagent kit (Beyotime, China), and cytokine levels (TNF-*α*, IL-6, IL-1β, IL-4, and IL-10) in the supernatant were measured using ELIZA kits (Beijing Quanshijin Biotechnology Co., Ltd., Beijing, China). Intracellular reactive oxygen species (ROS) were detected using the DCFH-DA fluorescent probe (MCE, USA), and fluorescence signals were visualized with the Dragonfly confocal system and quantified using ImageJ software.

Lysosomal Detection by Confocal Microscopy and Flow Cytometry. Lysosomal abundance and function were assessed using LysoTracker Green (Beyotime, China, C1047S) staining. After EV and LPS co-treatment, cells were stained and either imaged by confocal microscopy or analyzed by flow cytometry (Beckman Colter MoFlo Astrios, USA). To examine lysosome-dependent regulation, a subset of cells was pretreated with chloroquine (CQ, 20 μM; MCE, USA) for 4 h before EV addition to inhibit lysosomal acidification. For imaging assays, PKH26-labeled EVs and DAPI-stained nuclei were visualized using the Dragonfly spinning disk confocal microscope, and lysosomal fluorescence intensity was quantified by ImageJ.^54^

Quantitative Real-Time PCR (qPCR). Total RNA was extracted using the Total RNA Extraction Kit (Beijing Quanshijin Biotechnology Co., Ltd., Beijing, China), and cDNA was synthesized with the First-Strand cDNA Synthesis Kit (Beijing Quanshijin Biotechnology). Quantitative PCR was conducted using SYBR Green Supermix (Beijing Quanshijin Biotechnology) on a CFX96 Real-Time PCR System (Bio-Rad, USA). Amplification conditions were 95 °C for 2 min, followed by 40 cycles of 95 °C for 15 s and 60 °C for 30 s. Specificity was verified by melting curve analysis. Relative gene expression was normalized to GAPDH using the 2^−ΔΔCt^ method. Primer sequences are listed in Table 1.^55^

**Table 1.** Primer sequences used for quantitative real-time PCR (mouse genes).

Gene	Forward primer (5′ to 3′)	Reverse primer (5′ to 3′)
*mTOR*	AGAGGTCGGCACTCCACTAT	ATGTTCTGAGTCCCTGCTGC
*TFEB*	TGATGGGGAAGAACAGGGGT	GGGAGAGCTGTTAGCACCAA
*p62*	GACAAATATACCGGGGGCGG	AGCTGGTTTCGGGTGTGAAA
*LC3B*	TGACCCAGCTTAAGCGACTG	AACCACATCCTAAGGCCAGC
*LAMP1*	AGCATACCGGTGTGTCAGTG	GTTGGGGAAGGTCCATCCTG
*LAMP2*	AGGAGCCGTTCAGTCCAATG	GTGTGTCGCCTTGTCAGGTA
*GAPDH*	GAAGGCTATAGTCACCTCGGG	ATGGTAATAATGCGGCCGGT

Western Blot Analysis. Proteins were extracted using the RIPA Lysis Buffer Kit (Proteintech Group, Wuhan, China) supplemented with protease and phosphatase inhibitors. Protein concentrations were determined using the BCA Protein Assay Kit (Proteintech Group, Wuhan, China). Equal amounts of total protein (20 μg) were separated by SDS-PAGE and transferred onto PVDF membranes (Millipore, USA). Membranes were blocked with 5% nonfat milk in TBST for 1 h at room temperature, followed by incubation overnight at 4 °C with specific primary antibodies (Table 2). Primary and secondary antibodies were used at the manufacturer-recommended dilutions. After washing, membranes were incubated with HRP-conjugated secondary antibodies for 1 h at room temperature. Protein bands were visualized using an enhanced chemiluminescence (ECL) detection system (Thermo Fisher Scientific, USA). Band intensity was quantified using ImageJ software.^56^

**Table 2.** List of primary and secondary antibodies used in this study.

Antibody	Source	Application
Anti-mTOR (Rabbit)	Proteintech Group, Cat#81670-1-RR	WB
Anti-TFEB (Rabbit)	Proteintech Group, Cat#13372-1-AP	WB, IHC, IF
Anti-p62 (Rabbit)	Proteintech Group, Cat#84826-1-RR	WB
Anti-LC3B (Rabbit)	Proteintech Group, Cat#18725-1-AP	WB
Anti-LAMP1 (Rabbit)	Abcam, Cat#Ab320851	WB
Anti-LAMP2 (Rabbit)	Proteintech Group, Cat#32148-1-AP	WB
Anti-ZO-1 (Rabbit)	Proteintech Group, Cat#21773-1-AP	WB, IF
Anti-Occludin (Rabbit)	Proteintech Group, Cat#27260-1-AP	WB, IF
Anti-Claudin 1 (Rabbit)	Proteintech Group, Cat#28674-1-AP	WB
Anti-Ly6g Rabbit pAb	Servicebio, Cat#GB11229-50	IHC
Anti-F4/80 Rabbit pAb	Servicebio, Cat#GB113373-50	IHC
GAPDH Mouse McAb	Proteintech Group, Cat#60004-1-Ig	WB
HRP-conjugated Goat anti-Rabbit IgG	Proteintech Group, Cat#SA00001-2	WB, IHC
HRP-conjugated Goat anti-Mouse IgG	Proteintech Group, Cat#SA00001-1	WB, IHC
Goat anti-Rabbit IgG-Alexa Fluor 488	Abcam, Cat#ab150077	IF
Goat anti-Rabbit IgG-Alexa Fluor 594	Abcam, Cat#ab150080	IF

### 5.7. Animal experiments

Experimental Design and DSS-Induced Colitis Model. The DSS-induced colitis experiment comprised six groups, with six mice allocated to each group (36 mice in total). Eight-week-old male C57BL/6 mice were maintained under the housing conditions described above. Mice were allocated by simple randomization to the following groups: control (PBS), DSS only, low-dose LrEVs, high-dose LrEVs, low-dose ScEV-LrEVs, and high-dose ScEV-LrEVs. From day 1 to day 15, mice received a daily oral gavage of 200 µL of PBS or the corresponding vesicle suspension. The low- and high-dose EV groups received vesicle suspensions at concentrations of 1 × 10^10^ and 1 × 10^11^ particles/mL, respectively. The control and DSS-only groups received an equal volume of PBS. From day 8 to day 14, all groups except the control group received 3% dextran sulfate sodium (DSS; MP Biomedicals, 160110, molecular weight 36,000–50,000 Da) in the drinking water to induce colitis, whereas the control group received regular drinking water throughout the experimental period. Body weight, stool consistency, and rectal bleeding were recorded daily to calculate the disease activity index (DAI). Day 15 was defined as the terminal experimental endpoint. Following the final oral administration on day 15, all mice were euthanized under deep isoflurane anesthesia, and blood, colon, and spleen samples were collected for subsequent analyzes. Terminal outcome measures included colon length, spleen weight, histopathological changes, serum cytokine levels, inflammatory-cell infiltration, epithelial barrier markers, and lysosome-autophagy-related molecular markers. All animals completed the study and were included in the final analyzes.^57^

Histopathological Analysis (H&E Staining). Distal colon segments were fixed in 4% paraformaldehyde, embedded in paraffin, and sectioned at 5 μm thickness. Sections were deparaffinized, rehydrated, and stained with hematoxylin and eosin (H&E) following standard protocols. Histopathological scoring was performed under a light microscope (Leica DM2500, Germany) based on epithelial integrity, crypt structure, and immune infiltration.^58^

Serum Cytokine Measurement. Blood samples were collected by cardiac puncture at euthanasia and centrifuged at 3,000 × g for 15 min to obtain serum. The concentrations of TNF-*α*, IL-6, IL-1β, and IL-10 were determined using ELIZA kits (Beijing Quanshijin Biotechnology Co., Ltd., Beijing, China) according to the manufacturer’s instructions.

Immunohistochemistry (IHC). Paraffin-embedded colon sections were deparaffinized, rehydrated, subjected to antigen retrieval, and blocked with 3% hydrogen peroxide followed by 3% BSA. Sections were incubated overnight at 4 °C with primary antibodies against Ly6G (neutrophil marker) and F4/80 (macrophage marker) (Table 2), followed by incubation with HRP-conjugated secondary antibodies. Immunoreactivity was visualized using a DAB chromogenic substrate, counterstained with hematoxylin, dehydrated, and mounted. Positive staining was analyzed using ImageJ software.^59^

Immunofluorescence (IF) Analysis of Tight Junction Proteins. Colon sections were processed similarly for antigen retrieval and blocking, followed by incubation with primary antibodies against ZO-1 and Occludin (Table 2). After washing, sections were incubated with fluorophore-conjugated secondary antibodies and counterstained with DAPI (Beyotime, China). Images were captured with a Dragonfly spinning disk confocal microscope (Oxford Instruments, UK), and fluorescence intensity was quantified using ImageJ.^60^

TFEB Immunohistochemistry and Immunofluorescence. TFEB expression and localization in colonic tissues were evaluated using both IHC and IF as described above. For IHC, HRP detection was employed, while for IF, Alexa Fluor-conjugated secondary antibodies (Abcam, UK) were used, followed by nuclear counterstaining with DAPI.^61^

qPCR and Western Blot Analysis of Colonic Tissue. Total RNA was extracted from mouse colon tissues using the Animal Total RNA Isolation Kit (Beijing Quanshijin Biotechnology Co., Ltd., Beijing, China), and cDNA synthesis was performed with the Reverse Transcription Kit (Beijing Quanshijin Biotechnology Co., Ltd.). Quantitative PCR was conducted using SYBR Green Supermix on a QuantStudio 5 system (Applied Biosystems, USA). Primer sequences are listed in Table 1. For protein analysis, colonic tissues were homogenized and lysed using the Animal Total Protein Extraction Kit (Proteintech Group, Wuhan, China). Western blotting procedures were identical to those described in the cellular experiments, employing the same antibodies and detection methods listed in Table 2.

### 5.8. Comparative proteomic and lipidomic analyzes of LrEVs and ScEV-LrEVs

Proteomic Analysis. Extracellular vesicles isolated from *Lactobacillus rhamnosus* cultured without or with prior ScEV conditioning were designated LrEVs and ScEV-LrEVs, respectively, and subjected to label-free quantitative proteomic analysis. Vesicle proteins were extracted using RIPA lysis buffer supplemented with protease inhibitors and subjected to tryptic digestion. The resulting peptides were analyzed using an EASY-nLC 1200 system coupled to a Q Exactive HF-X Orbitrap mass spectrometer (Thermo Fisher Scientific, USA). Raw MS data were processed using MaxQuant (version 2.0) against the *L. rhamnosus* UniProt protein database. Principal component analysis (PCA) was performed to evaluate overall sample variation and separation between groups. Differentially abundant proteins were identified using thresholds of |log₂FC| ≥ 1 and *p* < 0.05 and visualized using volcano plots and hierarchical clustering heatmaps. Functional annotation and pathway analysis of the identified proteins were performed using the Gene Ontology (GO), Clusters of Orthologous Groups (COG), and Kyoto Encyclopedia of Genes and Genomes (KEGG) databases.

Lipidomic Analysis. For comparative lipidomic profiling, total lipids were extracted from LrEVs and ScEV-LrEVs using a methanol-based extraction method and analyzed by ultra-performance liquid chromatography coupled with tandem mass spectrometry (UPLC-MS/MS) using a QTRAP 6500 + system (AB Sciex, USA) in both positive- and negative-ion modes. The acquired data were processed using LipidSearch version 4.2 for lipid identification, peak alignment, and relative quantification. Principal component analysis and hierarchical clustering were performed to assess sample variability and group separation. Differentially abundant lipids were identified using thresholds of |log₂FC| ≥ 1 and *p* < 0.05 and visualized using volcano plots and heatmaps. Lipid subclass composition was summarized according to lipid category, and KEGG pathway enrichment analysis was performed to identify metabolic pathways associated with the lipidomic alterations.^46^

### 5.9. Loading of Cer(t18:0/24:0(2OH)) into LrEVs and cellular validation

Ceramide loading into extracellular vesicles. Based on prior studies of ceramide-modulated EV biogenesis and cargo loading,^62^ ^,^ ^63^ we adapted a direct incubation/ultracentrifugation method to incorporate Cer(t18:0/24:0(2OH)) into LrEVs. Cer(t18:0/24:0(2OH)) (C24; purity >95%; LIPID MAPS ID: LMSP02030002) was custom synthesized by Shanghai Hongye Biotech Co., Ltd. (Shanghai, China). The compound was dissolved in ethanol and dimethyl sulfoxide (DMSO) to prepare the working solution. Purified LrEVs were incubated with varying concentrations of ceramide at 37 °C for 15 min under gentle agitation. The concentration range used for C24 loading was selected based on preliminary cytotoxicity screening of free C24 and C24-loaded LrEVs, with subsequent functional assays conducted within the non-cytotoxic range. For subsequent functional assays, C24-LrEVs were prepared using C24 input concentrations of 0.2, 0.5, or 1 μM. These values represent the concentrations of C24 added to the loading mixture rather than the amounts ultimately incorporated into the recovered EVs. Fatty acid-free bovine serum albumin (BSA; Solarbio Life Sciences, Beijing, China) was added to enhance lipid dispersion. Free ceramide was removed by ultracentrifugation at 150,000 × g for 1.5 h at 4 °C, and the resulting ceramide-loaded LrEVs (C24-LrEVs) were resuspended in PBS for subsequent experiments.

Cellular validation. RAW264.7 macrophages were treated with free C24 (0.5 μM), C24-LrEVs prepared using C24 input concentrations of 0.2, 0.5, or 1 μM, native LrEVs, or ScEV-LrEVs. All EV preparations were normalized to 1 × 10^10^ particles/mL, and the corresponding vehicle controls were included. Cytotoxicity, nitric oxide (NO) production, cytokine secretion, and lysosome pathway-related protein expression were evaluated using the same procedures described in the Cellular Experiments section.

### 5.10. Data analysis

Quantitative data are presented as the mean ± standard deviation (SD). Statistical analyzes were performed using GraphPad Prism 10.0 (GraphPad Software, USA). Normality was assessed using the Shapiro-Wilk test. Comparisons between two groups were performed using an unpaired two-tailed Student’s t-test. Comparisons among three or more groups were performed using one-way analysis of variance followed by Tukey’s multiple-comparisons test. Longitudinal body-weight and disease activity index data were analyzed using two-way repeated-measures analysis of variance followed by Tukey’s multiple-comparisons test. When data did not satisfy the assumptions of parametric analysis, the Mann-Whitney U test was used for comparisons between two groups, whereas the Kruskal-Wallis test followed by Dunn’s multiple-comparisons test was used for comparisons among multiple groups. Statistical tests used for individual datasets are specified in the corresponding figure legends. Differences were considered statistically significant at *p* < 0.05. Unless otherwise stated, all experiments included at least three independent biological replicates.

## Supplementary Material

Clean files_Supplementary_material_R1.docx

## Data Availability

The raw 16S rRNA gene amplicon sequencing data generated in this study, including the *in vitro* human fecal microbiota experiment and the short- and long-term mouse experiments, are publicly available in the NCBI Sequence Read Archive under BioProject accession number PRJNA1498317 (https://www.ncbi.nlm.nih.gov/bioproject/PRJNA1498317). The proteomic and lipidomic abundance matrices, together with the GraphPad Prism project files containing the numerical source data and statistical analyzes underlying the figures, are publicly available on Zenodo at https://doi.org/10.5281/zenodo.21472799. All other data supporting the findings of this study are included in the article and its Supplementary Material.
