# Supplementary material for: Fungal extracellular vesicles reshape Lactobacillus rhamnosus vesicles to enhance lysosomal homeostasis and resolve intestinal inflammation
Source: Gut Microbes. 2026 Sep 19;18(1):2736915. doi: 10.1080/19490976.2026.2736915 (PMC13596881; doi:10.1080/19490976.2026.2736915)
Supplement: Supplementary file 1 [file KGMI_A_2736915_SM9025.docx]

**Fungal extracellular vesicles reshape *Lactobacillus rhamnosus* vesicles to enhance lysosomal homeostasis and resolve intestinal inflammation**

Zhengmei Ji ^a b c^, Guoliang Chen ^d^, Xiaolong He ^d^, Xiangqian Zhang ^d^, Bin Chen ^e^, Hang Xiao ^e^, Yurong Guo ^a b c d^ ,Yanhui Han ^a b c *^

^a^ Engineering Research Center for High-Valued Utilization of Fruit Resources in Western China, Ministry of Education, Shaanxi Normal University, 620 West Changan Avenue, Changan, Xi’an 710119, PR China

^b^ National Research & Development Center of Apple Processing Technology, Shaanxi Normal University, 620 West Changan Avenue, Changan, Xi’an 710119, PR China

^c^ College of Food Engineering and Nutritional Science, Shaanxi Normal University, 620 West Changan Avenue, Changan, Xi’an 710119, PR China

^d^ Research and Development Centre of Ecological and Sustainable application of Microbial Industry of the Loess Plateau in Shaanxi Province, College of Life Science, Yan'an University, Yan'an, Shaanxi, 716000, PR China

^e^ Department of Food Science, University of Massachusetts, Amherst, Massachusetts 01003, United States

^*^ Corresponding author: Yanhui Han

College of Food Engineering and Nutritional Science, Shaanxi Normal University,

620 West Changan Avenue, Changan, Xi’an 710119, P. R. China.

Tel.: +086 029 85310517, Fax: +086 029 85310517

E-mail: [yanhuihan@snnu.edu.cn](mailto:yanhuihan@snnu.edu.cn)

## Figure S1. Replicate particle-size distribution profiles and proteomic characterization of *Schizophyllum commune*-derived extracellular vesicles (ScEVs). (A) Intensity-based DLS size-distribution curves of ScEVs from three independent biological preparations. (B) Concentration-based NTA size-distribution curves of ScEVs from three independent biological preparations. Peak diameters are indicated in the plots. (C) Summary of identified and quantified proteins and peptides in ScEVs based on LC-MS/MS analysis (n = 3 independent ScEV preparations). (D) Gene Ontology (GO) annotation of identified proteins categorized into biological process (BP), cellular component (CC), and molecular function (MF) terms. (E) Clusters of Orthologous Groups (COG) functional classification of ScEV proteins. (F) KEGG pathway enrichment analysis of identified proteins. Quantitative data are presented as mean ± SD, and statistical analyses were performed as described in the main methods.

## Figure S2. Phylum- and genus-level taxonomic profiles associated with ScEV exposure. (A, B) Phylum- and genus-level composition of ScEV-positive bacterial populations after 6 h of in vitro incubation with human fecal microbiota. (C, D) Phylum- and genus-level composition of ScEV-associated bacterial populations 3 h after mice were orally gavaged with 200 μL of PKH26-labeled ScEV suspension (1 × 10^10^ particles/mL, corresponding to 2 × 10^9^ particles per mouse). (E, F) Phylum- and genus-level composition of fecal microbiota after mice received 200 μL of ScEV suspension (1 × 10^10^ particles/mL, corresponding to 2 × 10^9^ particles per mouse) by daily oral gavage for 30 consecutive days. For the 6 h in vitro experiment, fecal samples from nine donors were pooled to generate three composite samples per group, with each composite sample comprising material from three donors. For the 3 h in vivo experiment, fecal samples from nine mice were pooled to generate three composite sequencing samples per group, with each composite sample comprising material from three mice. The 30-day experiment included six mice per group. Taxonomic profiles are presented as relative abundance, and Lactobacillus-related taxa are highlighted by red boxes. The profiles in (A–D) indicate bacterial taxa associated with ScEV-positive signals or ScEV interaction and should not be interpreted as changes in absolute bacterial abundance.

## Figure S3. Uptake of ScEVs by target *Lactobacillus* species and particle size characterization of native and ScEV-conditioned extracellular vesicles. (A) Representative fluorescence microscopy images showing the association of PKH26-labeled ScEVs with *Lactobacillus rhamnosus* and *Lactobacillus reuteri* after 6 h of co-incubation. Insets show magnified views of the boxed regions (scale bar, 20 μm; representative images from n = 3 independent co-incubations). (B-E) NTA showing the particle size distribution of extracellular vesicles derived from *L. rhamnosus* (LrEVs), ScEV-conditioned *L. rhamnosus* (ScEV-LrEVs), *L. reuteri* (LreEVs), and ScEV-conditioned *L. reuteri* (ScEV-LreEVs) (n = 3 independent EV preparations per group). (F) Growth curves of *L. reuteri* cultured with or without ScEVs, monitored by optical density at 600 nm (OD_600_) (n = 3 independent cultures). (G) NTA showing increased concentrations of EVs secreted by *L. reuteri* after ScEV exposure (n = 3 independent EV preparations). Quantitative data are presented as mean ± SD, and statistical analyses were performed as described in the main methods.

## Figure S4. Uptake, cytotoxicity, and anti-inflammatory profiles of *L. rhamnosus-* and *L. reuteri-derived* EVs. (A) Representative confocal microscopy images showing uptake of PKH26-labeled LrEVs, ScEV-LrEVs, LreEVs, and ScEV-LreEVs after 6 h of incubation (scale bar, 20 μm; representative images from n = 3 independent experiments). (B-C) Cell viability of RAW264.7 cells treated with increasing EV concentrations (10^6^-10^11^ particles/mL) for 24 h, assessed by CCK-8 assay (n = 3 independent experiments). (D) Effects of LrEVs, ScEV-LrEVs, LreEVs, and ScEV-LreEVs on LPS-induced nitric oxide production and cytokine secretion (IL-6, TNF-α, IL-1β, IL-4, and IL-10) (n = 3 independent experiments). Quantitative data are presented as mean ± SD, and statistical analyses were performed as described in the main methods.

## Figure S5. ScEV-conditioned EVs from L. rhamnosus and L. reuteri attenuate ROS production in LPS-stimulated macrophages. (A) Representative fluorescence microscopy images showing intracellular reactive oxygen species (ROS) in LPS-stimulated RAW264.7 macrophages after treatment with LrEVs, ScEV-LrEVs, LreEVs, or ScEV-LreEVs (scale bar, 100 μm; representative images from n = 3 independent experiments). (B) Quantification of ROS fluorescence intensity (n = 3 independent experiments; 6 technical replicates per condition in each experiment). Quantitative data are presented as mean ± SD, and statistical analyses were performed as described in the main methods.

## Figure S6. Immunohistochemical analysis of TFEB expression in colon tissues of DSS-induced colitis mice treated with LrEVs and ScEV-LrEVs. (A) Representative immunohistochemical (IHC) staining images showing TFEB expression in colon tissue sections from different treatment groups (scale bars: 100 μm for low magnification, 20 μm for high magnification). (B) Quantification of TFEB staining intensity across groups, expressed as relative mean optical density (n = 6 mice per group). Quantitative data are presented as mean ± SD, and statistical analyses were performed as described in the main methods.

## Figure S7. Proteomic profiling reveals distinct protein composition between native LrEVs and ScEV-LrEVs. (A) Numbers of identified proteins and precursor peptides in native LrEVs and ScEV-LrEVs across three independent EV preparations (n = 3). (B) Principal component analysis (PCA) showing clear separation between LrEVs and ScEV-LrEVs, indicating distinct proteomic profiles (n = 3). (C) Volcano plot of differentially expressed proteins between LrEVs and ScEV-LrEVs, with significantly upregulated and downregulated proteins highlighted. (D) Gene Ontology (GO) enrichment analysis of differentially expressed proteins, classified into biological process (BP), cellular component (CC), and molecular function (MF) categories. (E) Kyoto Encyclopedia of Genes and Genomes (KEGG) pathway enrichment analysis of differentially expressed proteins. Quantitative data are presented as mean ± SD, and statistical analyses were performed as described in the main methods.

## Figure S8. Differentially downregulated lipid species between LrEVs and ScEV-LrEVs. (A) Top 15 downregulated lipid species in ScEV-LrEVs compared with LrEVs (n = 3 independent EV preparations per group). (B) Hierarchical clustering heatmap of the top 15 downregulated lipids, showing consistent reductions across replicates (n = 3 independent EV preparations per group). Quantitative data are presented as mean ± SD, and statistical analyses were performed as described in the main methods.

## Figure S9. Cytotoxicity assessment of free Cer(t18:0/24:0(2OH)) and Cer(t18:0/24:0(2OH))-loaded LrEVs in RAW264.7 macrophages. (A) Cell viability of RAW264.7 macrophages treated with increasing concentrations of free C24 was evaluated using the CCK-8 assay after 24 h of incubation (n = 3 independent experiments). (B) Cell viability of RAW264.7 macrophages treated with 1 × 10^11^ particles/mL of LrEVs loaded with different concentrations of C24, assessed by CCK-8 assay (n = 3 independent experiments). Quantitative data are presented as mean ± SD, and statistical analyses were performed as described in the main methods.

Figure S1.

Figure S2.

Figure S3.

Figure S4.

Figure S5.

Figure S6.

Figure S7.

Figure S8.

Figure S9.
